# The potyviral silencing suppressor HCPro recruits and employs host ARGONAUTE1 in pro-viral functions

**DOI:** 10.1371/journal.ppat.1008965

**Published:** 2020-10-08

**Authors:** Maija Pollari, Swarnalok De, Aiming Wang, Kristiina Mäkinen

**Affiliations:** 1 University of Helsinki, Department of Microbiology, Viikki Plant Science Center, Helsinki, Finland; 2 London Research and Development Centre, Agriculture and Agri-Food Canada, London, Ontario, Canada; University of California, Davis Genome Center, UNITED STATES

## Abstract

In this study, we demonstrate a novel pro-viral role for the *Nicotiana benthamiana* ARGONAUTE 1 (AGO1) in potyvirus infection. AGO1 strongly enhanced potato virus A (PVA) particle production and benefited the infection when supplied in excess. We subsequently identified the potyviral silencing suppressor, helper-component protease (HCPro), as the recruiter of host AGO1. After the identification of a conserved AGO1-binding GW/WG motif in potyviral HCPros, we used site-directed mutagenesis to introduce a tryptophan-to-alanine change into the HCPro (HCPro^AG^) of PVA (PVA^AG^) and turnip mosaic virus (TuMV^AG^). AGO1 co-localization and co-immunoprecipitation with PVA HCPro was significantly reduced by the mutation suggesting the interaction was compromised. Although the mutation did not interfere with HCPro’s complementation or silencing suppression capacity, it nevertheless impaired virus particle accumulation and the systemic spread of both PVA and TuMV. Furthermore, we found that the HCPro-AGO1 interaction was important for AGO1’s association with the PVA coat protein. The coat protein was also more stable in wild type PVA infection than in PVA^AG^ infection. Based on these findings we suggest that potyviral HCPro recruits host AGO1 through its WG motif and engages AGO1 in the production of stable virus particles, which are required for an efficient systemic infection.

## Author summary

In the course of an infection viral proteins come regularly into contact with host factors. Knowledge of these virus-host interactions in plants is essential for understanding the progress of infections and for addressing challenges in the production of healthy plants, which is important for global food security. In this study we report that the potyviral Helper component proteinase (HCPro) recruits host ARGONAUTE1 protein and engages it in pro-viral functions. We found that the interaction of potato virus A HCPro with ARGONAUTE1 benefited the infection by promoting the stability and accumulation of virus particles. When HCPro was engineered to disrupt the interaction, we detected very few virus particles and observed that the mutated virus did not spread efficiently. Thus, we propose that the interaction between HCPro and ARGONAUTE1 has biological relevance as it is important for formation of stable particles and achieving optimal systemic infection.

## Introduction

In nature, plants are constantly challenged by a variety of biotic and abiotic stresses including virus infections. Plant viruses are a ubiquitous and diverse group of parasites that harnesses the host’s cellular components in the execution of viral functions including replication, translation and movement. Many plant viruses cause substantial losses in agriculture and are thus a significant risk to global food and feed production [1]. Understanding the importance of virus infections not only in model plants but also in crops and ecosystems has recently been highlighted as the grand challenge of plant virology [2].

To counter virus infections and other pathogens plants must recognize the threat and deploy their defenses accordingly. Vector transmission of the viruses overcome the primary physical barriers of a waxy cuticle and cell wall, and therefore further layers of defense responses aimed at eliminating, attenuating or containing infection are required [3, 4]. RNA silencing is a major conserved regulatory and antiviral mechanism, which has been extensively studied in plants (Recently reviewed in [5–7]). Thus, the key steps leading to RNA silencing are well known: ARGONAUTE-family (AGO) proteins employ short RNA (sRNA) molecules to selectively destroy or inhibit complementary RNAs resulting in the silencing of the target (reviewed in [8]). RNA silencing enables the plant to combat transgene or viral gene expression but it is also crucial for the regulation of endogenous gene expression for example during development [9].

AGO proteins are effectors of RNA silencing characterized by the presence of hallmark PAZ- and PIWI-domains responsible for RNA-binding capacity and slicer activity, respectively [10]. The plant AGO family is diverse and its members have acquired specialized functions beyond their typecast as effectors of RNA silencing [8]. AGO proteins can, for example, take part in DNA repair, methylation and chromatin remodeling [11–14]. The model plant *Arabidopsis thaliana (*hereafter *Arabidopsis)* has ten AGO genes (AGO1-10) which are grouped based on phylogeny and their preferred selection of sRNA cargoes [15–17]. *Arabidopsis* AGO1 was the first described ARGONAUTE protein and it became the founding member of the family [9]. Originally identified via mutations causing defects in leaf development, AGO1 activity was soon found to be important to the phenomenon of gene silencing [18]. Befitting a canonical AGO protein, AGO1 is an RNA slicer with the ability to use short RNAs as guides to target complementary RNAs either to cleavage or to translational repression [19, 20]. Because of their importance in RNA silencing, AGOs are traditionally placed at the frontline of host antiviral defense [3, 21, 22].

Potyviruses (family *Potyviridae*) are the largest genus of positive-sense single-stranded RNA viruses in plants [23, 24]. Because they are geographically widespread and infect numerous crops, potyviruses are ranked among the top ten economically most important plant viruses [1, 25]. Potyvirus genomes are compact, circa 10 kb in length. Their genome organization is conserved and encompasses two open reading frames, which code for eleven viral proteins [24]. The genomic RNA has a poly-A tail and contains a covalently attached viral protein genome-linked (VPg) at the 5’end. Potyviruses are encapsidated into flexible filamentous particles that are transmitted by aphid vectors in a non-persistent manner.

Many viral proteins are multifunctional, which compensates for their limited number. The potyvirus-specific helper component protease (HCPro) is a widely studied example of a multitasking viral protein (reviewed in [26]). In addition to acting as the major RNA silencing suppressor [27–29], it is involved in aphid-mediated transmission [30, 31], polyprotein maturation [32, 33], the assembly of virus particles [34, 35] and local and systemic spread [36–38]. Recently, HCPro has also been shown to be essential for the formation of potyvirus-induced granules, potential shelters for vRNA against the host’s silencing machinery [39].

Evidence accumulated over the past years has highlighted the relationships between viral proteins and plant AGOs. Although AGO2 has been established as the major antiviral AGO protein in leaves [40–43], AGO1 is still an important target of numerous viral silencing suppressors [44–53]. Forming a link to potyvirus infection, we have previously demonstrated the co-presence of AGO1 and the potato virus A (PVA) silencing suppressor HCPro on *Nicotiana benthamiana* (hereafter *N*. *benthamiana*) ribosomes during PVA infection [54]. This association suggests AGO1 could be involved in the repression of potyviral translation as part of host defense. Moreover, both proteins are integral components of potyvirus-induced granules [39]. Against this background, we set to investigate the relationship between AGO1 and HCPro and its prospective biological importance in more detail.

In this study, we show that the potyviral silencing suppressor HCPro interacts with AGO1 through a conserved WG motif and directs AGO1’s association with the viral coat protein CP. Furthermore, we report the relevance of the interaction for the systemic spread of the infection and suggest that AGO1 contributes to the success of potyvirus infection by enabling the accumulation of stable virions.

## Results

### AGO1 promotes PVA infection and is required for particle accumulation

**Fig 1:**
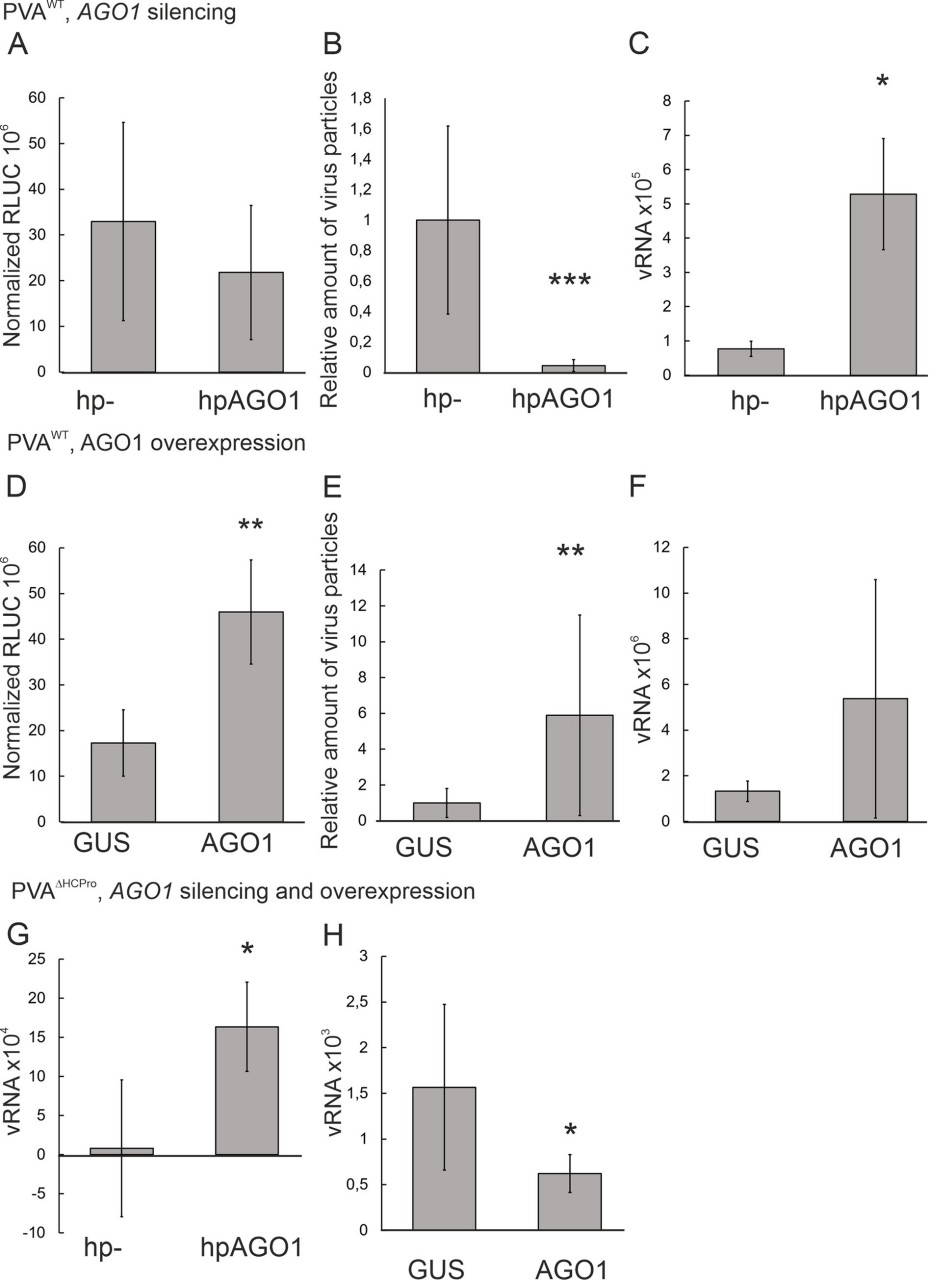
The availability of AGO1 has a significant impact on PVA infection and HCPro is responsible for taming AGO1’s antiviral effects. A-C) The effect of silencing of *AGO1* on PVA^WT^ gene expression (A), particle accumulation (B) and vRNA level (C) in locally infected leaves. D-F) The effect of AGO1 overexpression on PVA^WT^ gene expression (D), particle accumulation (E) and vRNA level (F) in locally infected leaves. G, H) The effect of silencing (G) and overexpression (H) on PVA^ΔHCPro^ vRNA levels in locally infected leaves. PVA^WT^ or PVA^ΔHCPro^ and firefly luciferase were co-infiltrated at OD_600_ 0.005 and 0.01, respectively with either the silencing constructs (OD_600_ 0.4) or the overexpression constructs (OD_600_ 0.3). Samples were taken from the infiltrated leaves at 5 dpi. Viral gene expression was measured with the dual luciferase assay after which PVA^WT^-derived *Renilla* luciferase activity was normalized to firefly luciferase activity. The abundance of encapsidated viral genomes was determined by IC RT-PCR and the level of viral RNA was likewise measured by RT-PCR. RLUC and immunocapture data is presented as means of at least three independent experiments. RNA levels are representative results of three independent experiments. See also S1 Fig for *AGO1* and *AGO2* mRNA expression levels. Error bars denote standard deviation and student’s t-test was used to calculate statistical significance (* P<0.05, ** P<0.01, *** P<0,001).

To clarify the role of AGO1 in potyvirus infection, we first studied the effects of AGO1 scarcity and surplus on the progress of PVA infection in *N*. *benthamiana*. The *AGO1* gene was transiently knocked down by expressing a hairpin construct targeting its mRNA in *N*. *benthamiana*. An empty pHELLSGATE vector (hp-) was used as the control. We used an infectious cDNA (icDNA) clone of PVA tagged with the *Renilla* luciferase (*RLUC*) gene for accurate quantitation of PVA gene expression [55]. Henceforth we refer to this virus as PVA^WT^. PVA^WT^ gene expression was determined by measuring virus-derived RLUC reporter activity from both *AGO1*-silenced and control samples. The values were normalized to firefly luciferase activity in the same samples. Immunocapture reverse transcription polymerase chain reaction (IC RT-PCR) was used to determine PVA^WT^ particle accumulation in the same samples. In IC RT-PCR capture of virus particles with anti-CP antibodies is followed by the quantification of enclosed vRNA. The vRNA copy number is used to describe the number of intact particles. Although silencing of *AGO1* did not significantly alter PVA^WT^ gene expression, it slightly increased the amount of vRNA compared to the control (Fig 1A and 1C) and drastically reduced PVA^WT^ particle accumulation (Fig 1B). The relative amount of PVA^WT^ RNA within particles in *AGO1*-silenced leaves was 50 fold lower than in the non-silenced controls (Fig 1B). On the other hand, an abundance of AGO1, achieved by transient overexpression, significantly enhanced both PVA^WT^ gene expression and particle accumulation. AGO1 overexpression improved PVA^WT^ gene expression by over two-fold and yielded six times more vRNA within particles compared to the control although vRNA levels were not significantly affected (Fig 1D–1F). To gain further information about the role of HCPro, we checked the effect of AGO1 silencing and overexpression on the vRNA levels of an HCPro-deficient virus (PVA^ΔHCPro^). Higher vRNA levels were measured in *AGO1* -silenced samples while an excess of AGO1 reduced the amount of vRNA (Fig 1G and 1H). The hairpin silencing system was judged to provide sufficient silencing of *AGO1* as its mRNA levels were less than 20% of those in the control as measured by qRT-PCR (S1A Fig). Expression of *AGO2* mRNA was increased in *AGO1* silenced plants implicating possible co-regulation in their functions (S1A Fig).

It was clear that while AGO1 targeted the HCPro-less virus in a defensive manner it has a positive effect on the infection in the presence of a functional HCPro. AGO1 was especially important for virus particle production as its knock-down nearly abolished particle accumulation.

### Identification and site-directed mutation of a putative ARGONAUTE-binding motif conserved in potyviral HCPros

HCPro is renowned for its capacity to interact with a wide range of host factors. As our results hinted AGO1 could benefit the infection instead of countering it, we were interested in exploring the mechanisms and viral factors involved. The potyviral silencing suppressor HCPro provided an obvious starting point as its absence sensitized the virus to AGO1 and we had earlier demonstrated its involvement with both viral translation and AGO1 association (Fig 1, [54]).

**Fig 2:**
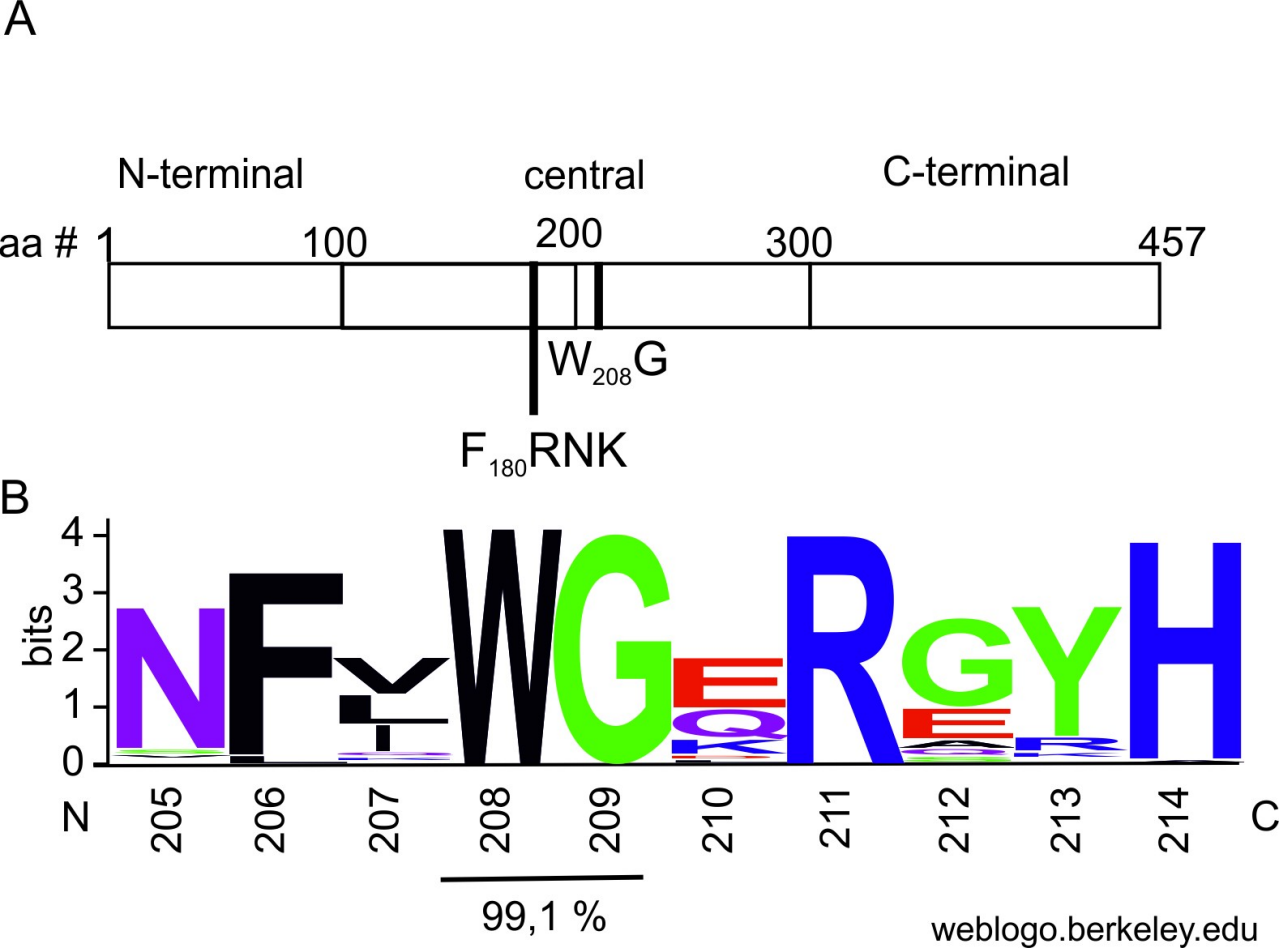
Potyviral HCPro sequences contain a conserved putative AGO-binding motif. A) Schematic diagram of potyviral HCPro showing the relative locations of the FRNK RNA-binding motif and the WG pair in the central region of HCPro.B) Conservation of the WG pair and surrounding amino acid residues in the HCPro’s of 113 potyvirus species. Amino acid residues are numbered according to the PVA HCPro sequence. See also S1 Table.

In the current study, we used a computational sequence analysis tool [56, 57] to search for putative WG/GW -type AGO1 binding domains in HCPro. The search revealed a single WG motif in the central region of PVA HCPro. This motif contains one WG pair at W208 G209 (Fig 2A). Although the motif was predicted to have low compositional compatibility to an ideal AGO-binding domain, a multiple sequence alignment of 113 potyviral polyproteins revealed the WG pair was conserved in 99.1% of the sequences (Fig 2B). Only banana bract mosaic virus (BBrMV, GenBank accession no. YP_001427389) lacked the WG pair (SI Table, S2 Fig). The high degree of conservation of the WG motif in HCPros throughout the genus *Potyvirus* spoke strongly in favor of its functional importance.

Next, we focused on deciphering the role of the WG motif in the context of the HCPro-AGO1 interaction and its potential significance in potyvirus infection. Site-directed mutagenesis was performed to change the tryptophan residue in the WG pair to alanine (W208A). Corresponding WG motifs in the icDNAs of two potyviruses, PVA and turnip mosaic virus (TuMV) were chosen as targets because this would allow us to compare and contrast the effects of the alanine substitution in two related virus species. The resulting versions with mutated HCPro sequences were named PVA^AG^ and TuMV^AG^, respectively. Additionally we generated Twin Strep-tag, RFP fusion constructs of PVA HCPro and HCPro^AG^ to facilitate their transient overexpression and detection in confocal fluorescence microscopy and co-immunopurification experiments. For the sake of clarity, these fusion proteins are called HCPro and HCPro^AG^.

**Fig 3:**
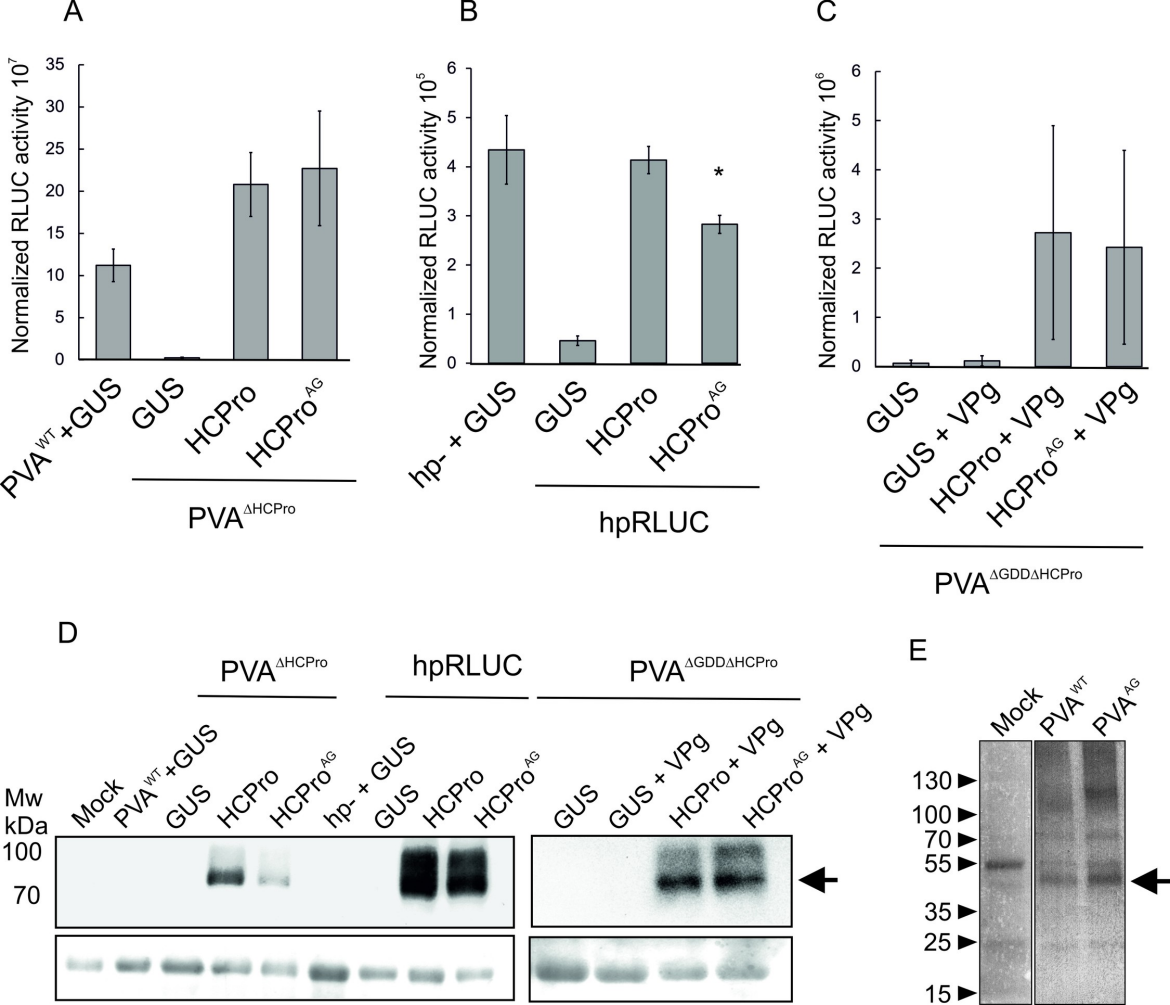
Basic functional characterization of HCPro^AG^. A) Complementation of the HCPro-less virus PVA^ΔHCPro^ by HCPro and HCPro^AG^. PVA^ΔHCPro^ and transient expression constructs for GUS, HCPro or HCPro^AG^ were co-infiltrated into *N*. *benthamiana* epidermal cells at OD_600_ 0.05 and 0.3, respectively. PVA^WT^ supplemented with GUS was used as the positive control. Firefly luciferase was included for normalization purposes in all sets at OD_600_ 0.01. Samples were taken at 5 dpi followed by a dual luciferase assay to determine virus-derived RLUC activity. B) Silencing suppression test. A hairpin construct targeting the *Renilla* luciferase mRNA (hpRLUC) or the empty vector (hp-) were infiltrated at OD_600_ 0.4 one day prior to the co-infiltration of RLUC and GUS, HCPro or HCPro^AG^ overexpression vectors each at OD_600_ 0.3. As above firefly luciferase was included in all sets at OD_600_ 0.01. Samples were taken at 4 dpi. C) Translational enhancement of PVA^ΔGDDΔHCPro^ by the coexpression of VPg and HCPro or HCPro^AG^. D) The transient expression of HCPro/HCPro^AG^ in the infiltrated leaves was confirmed by western blotting using a mouse monoclonal anti-RFP antibody (top panels). Arrow indicates the Twinstrep-tag RFP-HCPro fusion proteins. Equal loading of the gels was checked by staining the membranes with Ponceau solution (bottom panels). E) PVA^WT^- and PVA^AG^-infected leaf samples and a mock sample were subjected to a western blot analysis using anti-HCPro antibodies. The size of the monomeric HCPro is marked by an arrow. An unspecific band is visible in the mock sample, but it is slightly larger than HCPro.

The biological functionality of HCPro^AG^ was evaluated by analysing its complementation capacity by supplementing the HCPro-deficient virus (PVA^ΔHCPro^) with either HCPro, HCPro^AG^ or GUS as a negative control. Recovery of viral gene expression to the level of PVA^WT^ confirmed that HCPro^AG^ complemented the expression of PVA^ΔHCPro^ as well as the wild type protein (Fig 3A).

The main potyviral silencing suppressor HCPro is able to rescue the expression of genes targeted to silencing [28, 29]. When a hairpin construct was used to prime the plants for silencing *RLUC* mRNA, transient overexpression of HCPro^AG^ resulted in the nearly complete restoration of RLUC expression from the co-infiltrated monocistronic *RLUC* mRNA (Fig 3B). Altogether, the results suggested HCPro^AG^ suppressed silencing similarly to HCPro. Another of HCPro’s critical functions in PVA infection biology is to enable and enhance viral translation together with VPg [39]. We investigated the performance of HCPro^AG^ in the translational enhancement of a replication-deficient virus lacking HCPro (PVA^ΔGDDΔHCPro^). In this experiment, HCPro, HCPro^AG^ or a GUS control were co-expressed with VPg and PVA^ΔGDDΔHCPro^ translation was measured as virus-derived RLUC activity. Co-expression of VPg with either HCPro or HCPro^AG^ resulted in a circa 30 fold translational enhancement (Fig 3C). Expression of the GUS control alone or together with VPg did not significantly improve viral translation. Western blot analysis was used to confirm that HCPro and HCPro^AG^ were expressed at similar levels in the experiments (Fig 3D). Finally, another α-HCPro western blot was carried out from full length PVA^WT^ and PVA^AG^ infected samples to show presence of monomeric HCPro as evidence of intact auto-proteolytic activity of HCPro (Fig 3E).

Based on these results the disruption of the WG motif did not significantly disturb HCPro’s functionality in PVA expression. The mutated version of the protein retained its essential biological functions including complementation capacity, silencing suppression activity and the ability to enhance translation in collaboration with VPg.

### A disrupted WG motif compromises HCPro’s co-localization and interaction with AGO1

**Fig 4:**
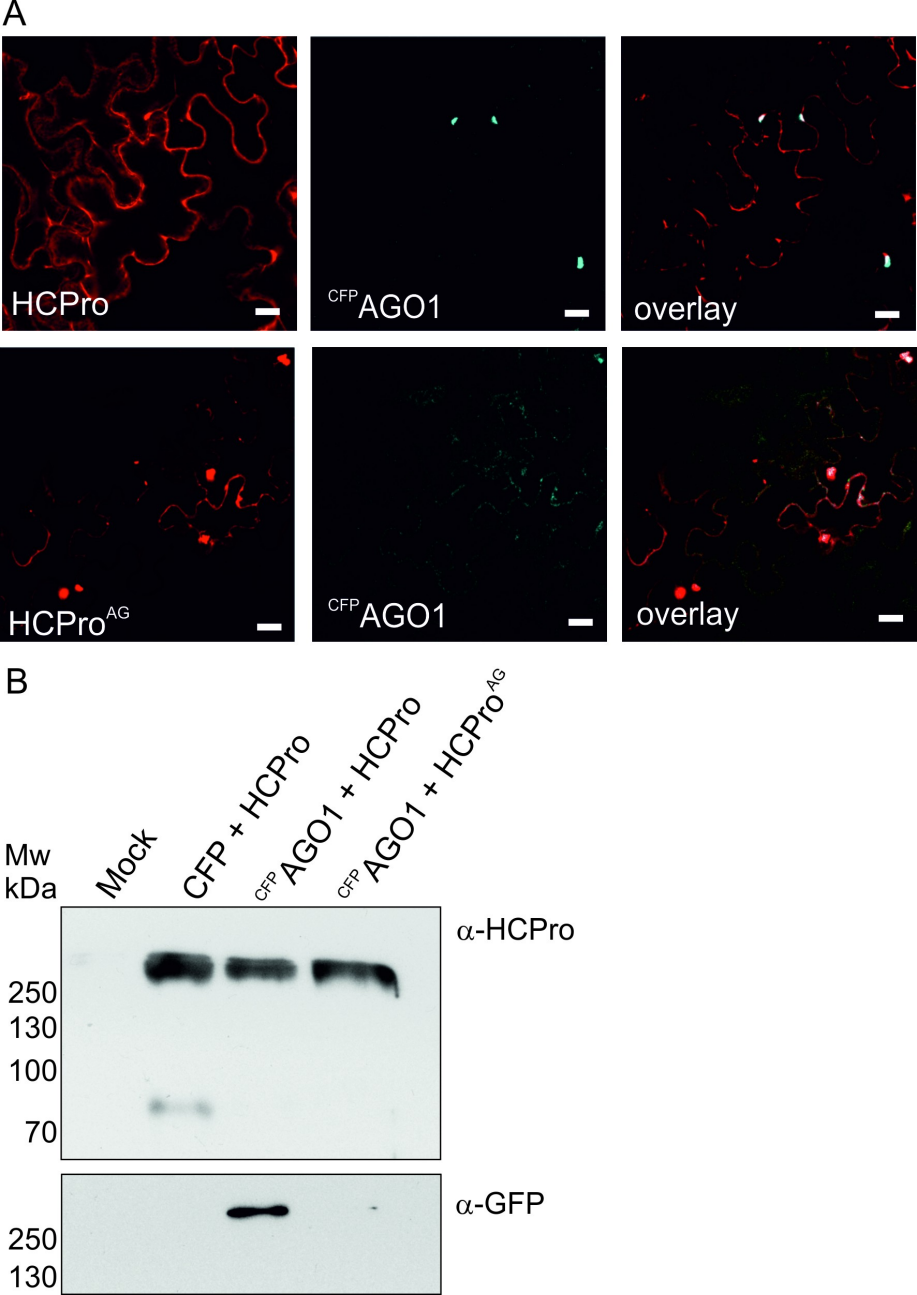
Disruption of the WG motif impairs AGO1 co-localization and co-immunopurification with HCPro. A) Co-localization of HCPro and HCPro^AG^ together with ^CFP^AGO1 in *N*. *benthamiana* leaves. All constructs were agroinfiltrated at OD_600_ 0.1 and leaves were imaged by confocal microscopy at 3 dpi. Representative images from three independent experiments are shown, scale bar is 20 μm. For control experiments with unfused CFP and RFP, see S3B Fig. ^CFP^AGO1 co-immunoprecipitates with HCPro but not with HCPro^AG^. Agroinfiltrations were done as in the confocal microscopy experiments and samples were taken at 3 dpi. Subsequently HCPro/HCpro^AG^ were affinity purified using MacroPrep strep tactin. Anti-HCPro and anti-GFP antibodies were used to detect HCPro/HCPro^AG^ and co-purified ^CFP^AGO1, respectively.

To study the intracellular localization of HCPro/HCPro^AG^ and ^CFP^AGO1 the expressed proteins were visualized in *N*. *benthamiana* by confocal microscopy. Unfused RFP and CFP proteins served as controls. At 3 dpi, the epidermal layer of cells was examined under a confocal microscope and fluorescence emission from RFP and CFP was detected in sequential scanning mode. Control experiments showed that in the absence of HCPro the ^CFP^AGO1 signal was weak and uniformly distributed in the cytoplasm (S3A Fig). In the presence of wild type HCPro, ^CFP^AGO1 fluorescence accumulated predominantly in granular structures that also contained HCPro (Fig 4A). Co-localization analysis revealed that the sequestration of ^CFP^AGO1 into HCPro-containing granules was significantly reduced but not completely abolished in the presence of the HCPro^AG^ mutant. The degree of ^CFP^AGO1 co-localization with HCPro^AG^ was reduced to 45 ± 38% from the 82 ± 18% of with the wild type protein (P-value of T-test 4 x 10^−9^; in 3 independent experiments) indicating that less ^CFP^AGO1 accumulated to granules formed by HCPro^AG^ than by HCPro.

Thus, according to confocal microscopy results, HCPro and ^CFP^AGO1 co-localized in *N*. *benthamiana* cells and the disruption of the WG motif interfered with the degree of co-localization. This observation supported the hypothesis that the motif could influence HCPro and AGO1 interaction.

The HCPro-AGO1 interaction was then studied by affinity purification/co-immunopurification using HCPro or HCPro^AG^ as baits. For the affinity purification experiments, we co-expressed the bait proteins with either ^CFP^AGO1 or CFP as a negative control. All constructs were agroinfiltrated into *N*. *benthamiana* plants at OD_600_ 0.1 and the infiltrated leaves were sampled at 3 dpi. Overexpression of AGO1 in mRNA level is validated in S3B Fig. We conducted affinity purification with cross-linked StrepTactin MacroPrep resin followed by western blotting with anti-HCPro and anti-GFP antibodies to detect the baits and ^CFP^AGO1 target, respectively. The majority of HCPro/HCPro^AG^ eluted in stable high molecular weight complexes that withstood SDS treatment and heat denaturation (Fig 4B). Some monomeric forms were also present as indicated by a band at ~75 kDa in the HCPro + CFP control sample. The western blots clearly showed ^CFP^AGO1 co-immunoprecipitated with HCPro but not with HCPro^AG^. The anti-GFP western blot detected ^CFP^AGO1 in high molecular weight complexes uniquely in association with the HCPro bait. ^CFP^AGO1 was not detected in the high molecular weight complexes when HCPro^AG^ was used as bait (Fig 4B). The specificity of the HCPro-AGO1 interaction was supported by the observation that the CFP control alone did not co-immunoprecipitate with HCPro (Fig 4B). The loss of ^CFP^AGO1 co-immunoprecipitation in HCPro^AG^ compared to the wild type protein indicates the capacity for AGO1 interaction was abolished or at least significantly weakened by the W208A mutation.

### Impaired systemic infection in PVA^AG^ and TuMV^AG^

We next assessed the effects of the W208A mutation on PVA and TuMV infections *in planta*. PVA^WT^ and PVA^AG^ were infiltrated into *N*. *benthamiana* at OD_600_ 0.05 and TuMV^WT^/TuMV^AG^ at OD_600_ 0.5. PVA gene expression was determined as RLUC activity using a dual luciferase assay and the accumulation of TuMV CP was determined with a DAS ELISA assay. Samples were obtained from local leaves at 3 dpi and from emerging systemic leaves at 8 or 14 dpi.

**Fig 5:**
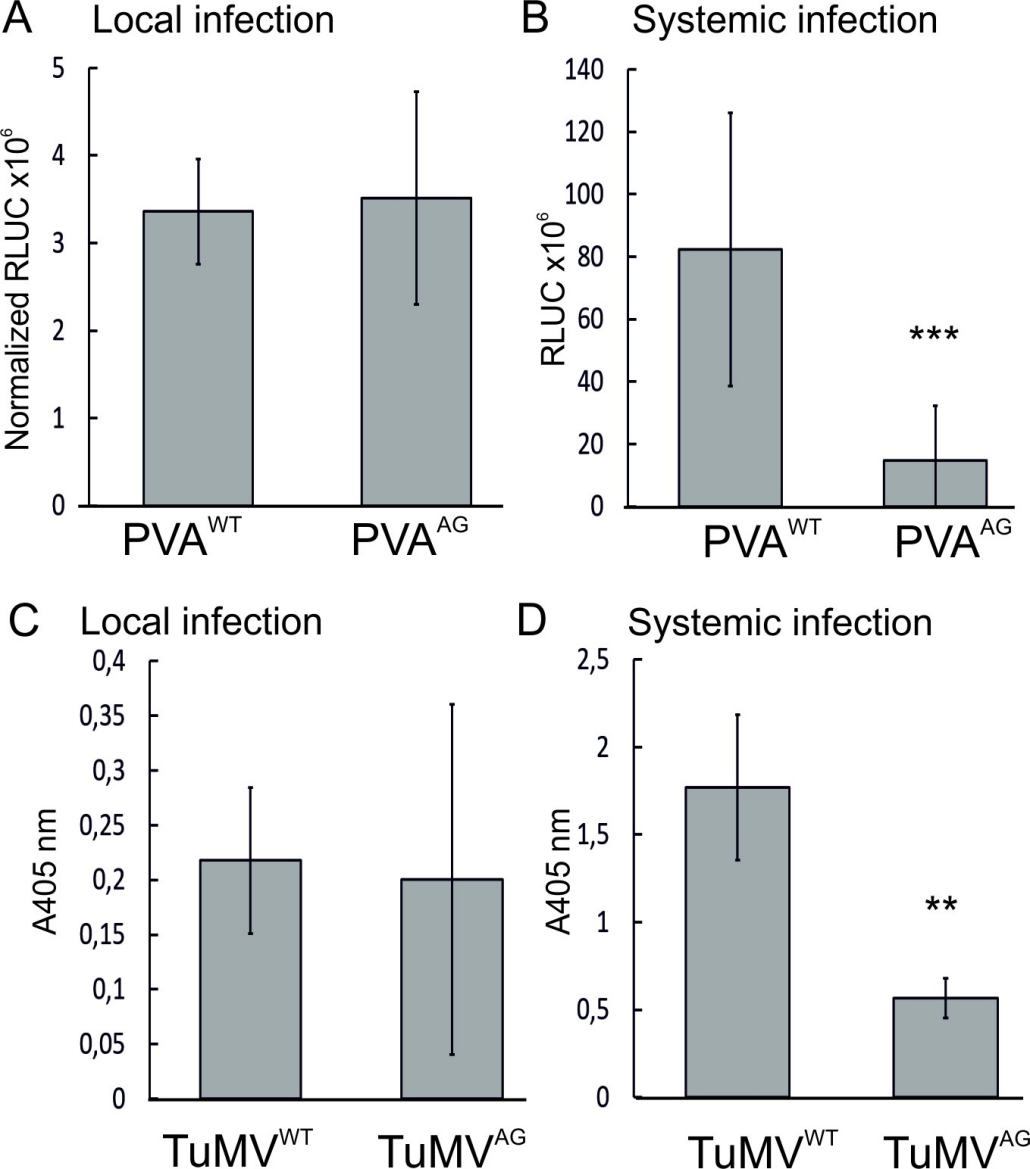
The W208A mutation impairs systemic infection in both PVA and TuMV. A, B) Effect of the mutation on local and systemic PVA infection. C, D) Effect of the mutation on local and systemic TuMV infection. PVA constructs were agroinfiltrated at OD_600_ 0.05 and TuMV constructs at OD_600_ 0.5. PVA and TuMV samples were taken from infiltrated leaves at 3 dpi and from young systemic leaves at 8 and 14 dpi, respectively. PVA gene expression was determined by measuring RLUC activity and TuMV CP accumulation was detected with a DAS ELISA assay. Results are averages of at least three independent experiments, error bars denote standard deviation and student’s t-test was used to calculate statistical significance (** P<0.01, *** P<0.001).

At 3 dpi, PVA^AG^ gene expression levels were similar to that of PVA^WT^ in infiltrated leaves. Likewise, there were no differences in CP accumulation measured from local leaves infiltrated with TuMV^WT^ and TuMV^AG^ (Fig 5A and 5C). As the dual reporter combination in the TuMV plasmids conveniently enables the tracking of viral cell-to-cell movement, we also compared the initial movement of TuMV^WT^ and TuMV^AG^ and found that the mutant spread to neighboring cells as efficiently as the wild type virus. The average number of neighboring cells infected by TuMV^WT^ was 2.5±2.4 and TuMV^AG^ 1.8±1.9 in three independent experiments. Error is denoted as standard deviation and the difference did not have any statistical significance. In the dual reporter system, primarily infected cells emit both mCherry and GFP fluorescence while secondarily infected cells only emit virus-derived GFP fluorescence. The viruses were infiltrated at a low OD_600_ of 0.005 to start the infection from individual, sparsely located cells and the leaves were then inspected under a confocal microscope at 3 dpi. Primarily infected epidermal cells were identified based on overlapping mCherry and GFP fluorescence and the number of infected neighboring cells emitting only GFP fluorescence was calculated.

Similar gene expression and cell-to-cell movement signified that the local infection processes of PVA and TuMV were undisturbed by the disruption of the AGO-binding motif in HCPro. However, when the infection was allowed to proceed for one week more, we found that the mutant viruses could not establish a systemic infection as well as the wild type viruses. PVA gene expression and TuMV CP accumulation in non-infiltrated systemic leaves was reduced in both PVA^AG^ and TuMV^AG^ compared to the wild type viruses. After 8 dpi PVA^AG^ gene expression reached only 20% of that of PVA^WT^ while TuMV^AG^ CP amount was, at 14 dpi, circa 30% of TuMV^WT^ (Fig 5B and 5D).

### Virus particle accumulation depends on the HCPro-AGO1 interaction

As silencing of *AGO1* nearly abolished the accumulation of virus particles, we decided to investigate if disruption of the HCPro-AGO1 interaction affected particle production. In many cases phloem-based long-distance spread of virus infections relies on virus particles [58]. Poor systemic infections by the mutant viruses indeed pointed us to this direction.

**Fig 6:**
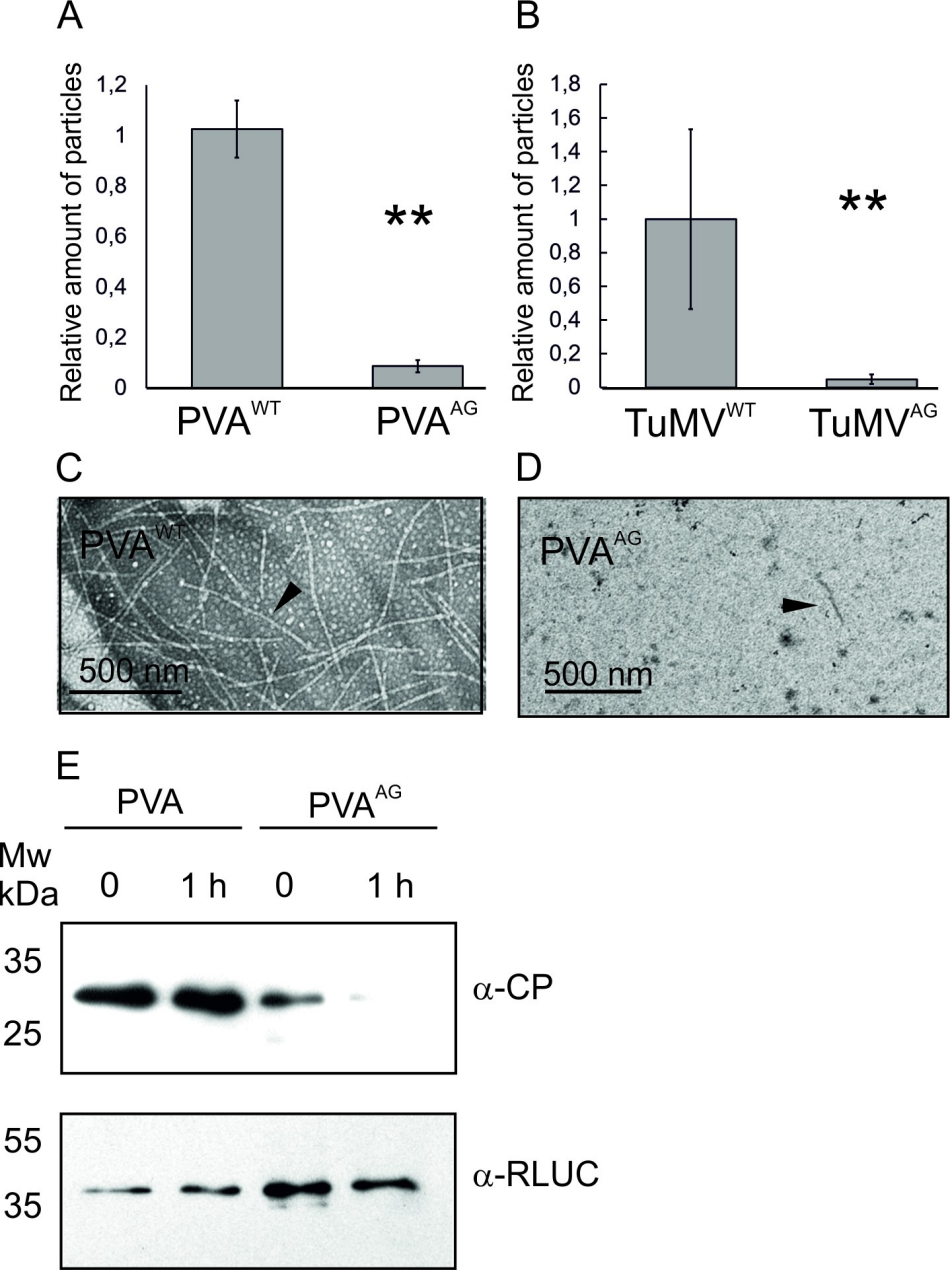
Accumulation of virus particles is disabled in PVA^AG^ and TuMV^AG^ and PVA CP becomes susceptible to degradation. A) Relative amounts of virus particles in systemic PVA^WT^/PVA^AG^ infection at 9 dpi. B) Relative amounts of virus particles in systemic TuMV^WT^/TuMV^AG^ infection at 12 dpi. The amount of particles was determined by IC RT-PCR and average results of three independent biological replicates are shown. Error bars denote standard deviation (** P<0.01). C, D) PVA^WT^ particles and virus-like particles from PVA^AG^, respectively, visualized with negative staining and transmission electron microscopy. Black arrows point to a representative particle in PVA^WT^ and a virus-like particle in PVA^AG^. E) Western blots showing the levels of CP and RLUC from PVA^WT^/PVA^AG^ samples at 0 h and after 1 h of incubation. Leaf samples were homogenized, suspended in 0.1 M Na-phosphate buffer pH 7.4 and the sap was incubated at room temperature for 1 h. Western blots were performed to detect CP (top panel) and RLUC (bottom panel).

The effect of the W208A mutation on PVA and TuMV particle production was studied by IC RT qPCR in *N*. *benthamiana* infected with either PVA^WT^/PVA^AG^ or TuMV^WT^/TuMV^AG^. In both PVA and TuMV the mutation had a drastic negative effect on virus particle accumulation. The relative amount of PVA^AG^ particles was, on average, only 9% of that of the wild type virus in systemically infected leaves (Fig 6A). Viral particle production was significantly reduced also in TuMV^AG^, which yielded 18-fold less particles than the wild type virus (Fig 6B).

Next, we compared the visual appearance of PVA^WT^ and PVA^AG^ -derived particles using transmission electron microscopy. Fully assembled virions were abundant in a PVA^WT^ infection at 9 dpi while only very few short and thin rod-shaped structures were observed in PVA^AG^ samples (Fig 6C and 6D). While these structures could represent virus-like particles, they are unlikely to contain full-length vRNA. They may instead be degraded or otherwise deformed particles.

### PVA CP is degraded faster in a PVA^AG^ infection than in the wild type PVA infection due to misassembled particles

The dramatic reduction in the amount of virus particles was intriguing since it was disproportionate compared to the modest decrease in virus-derived gene expression in systemic leaves (comparison with Fig 5B and 5D). Stability of the CP is another indicator of virion formation because free CP that is not assembled into particles is accessible to proteases. Based on the viral gene expression results (Fig 5) we deduced that both wild type and mutant viruses produce CP and hypothesized that if PVA^AG^ is unable to use the available CP in the production of stable viruses, the protein would be more susceptible to degradation. To test this idea we performed a CP stability assay on samples from PVA^WT^ and PVA^AG^ infections. Crude plant sap was incubated for one hour at room temperature followed by western blotting to detect CP and RLUC protein levels. Western blotting revealed the CP amount remained unaltered in the PVA^WT^ samples whereas CP produced by PVA^AG^ was almost completely degraded during the incubation period (Fig 6E). The result supported the idea that CP produced by PVA^AG^ was vulnerable because it was not incorporated into stable virions. Furthermore, the effect was CP-specific as the level of the RLUC reporter protein remained similar after the 1 h incubation.

**Fig 7:**
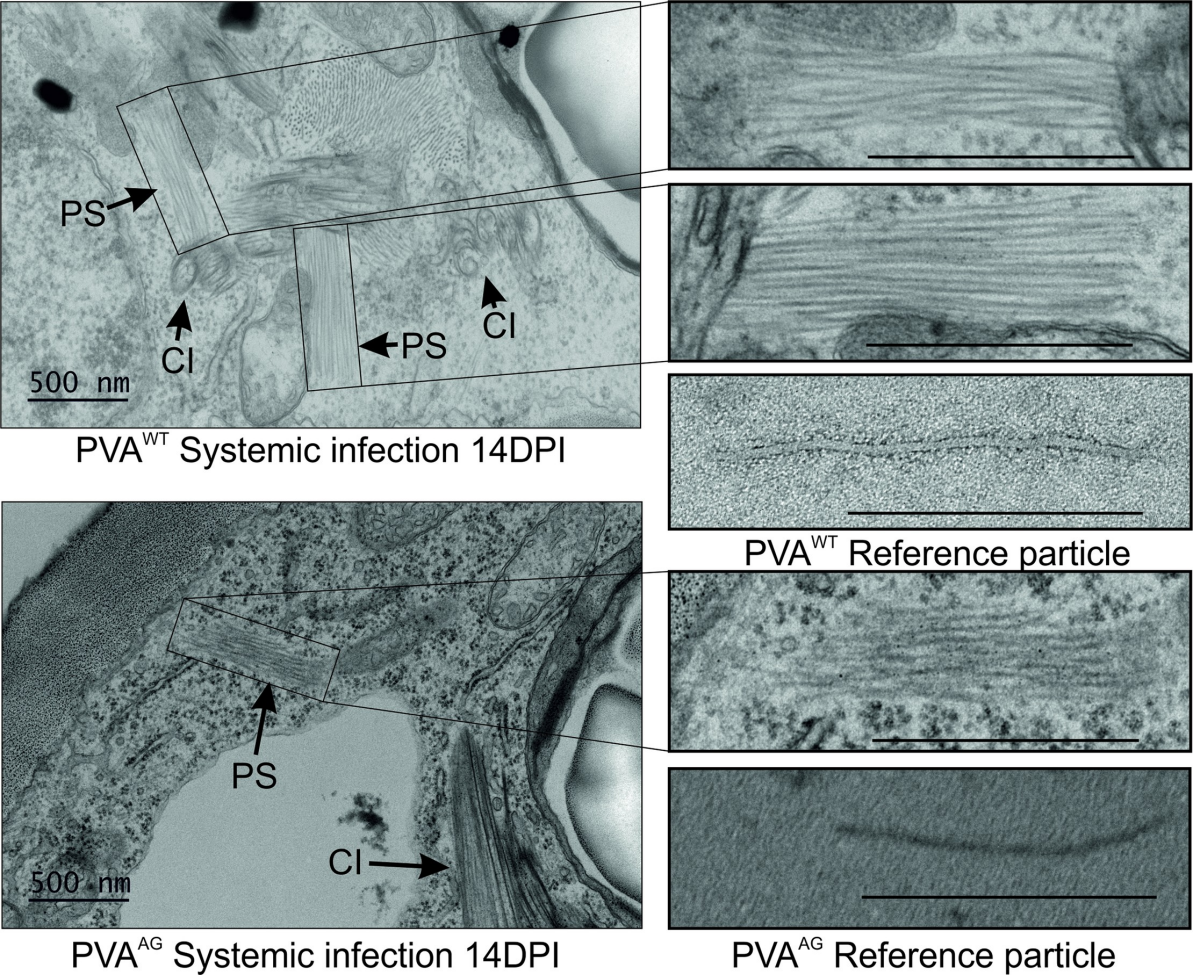
PVA^AG^ infection produces short and malformed virus particles. A) Both PVA^WT^ and PVA^AG^ infections were allowed to spread in the systemic leaves until 14 dpi. Thereafter, infected tissue sections were prepared and imaged by TEM. Both viruses generated ample pinwheel inclusions (marked as ‘CI’). However, those samples prepared from PVA^WT^ infected leaves (upper left panel) were always associated with stacks of particles (marked as ‘PS’) of standard size (upper right panel). Very few PVA^AG^ infected cells contained PS-like structures (lower left panel), however, upon magnification these structures revealed incomplete virus-like particles (lower right panel). Isolated PVA^WT^ particles and PVA^AG^ particle-like structures are given as a size reference below the magnified particle stacks. The bar is 500 nm.

Because IC-RT-PCR and electron microscopy of particles require the disruption of cells prior to analysis, we used transmission electron microscopy of thin sections of infected leaves to study particle formation in intact samples. This method revealed abundant stacks of particles in the vicinity of CI pinwheel structures in PVA^WT^ infected cells (Fig 7). The average length of the particles in PVA^WT^ infected tissues was around 700 nm. The accuracy of the estimation was somewhat impaired by the fact that the surrounding structures covered the exact ends of the stacked particles. Although some similar stacks were observed in PVA^AG^-infected leaves, they were detected only in a subset of infected cells. The stacked PVA^AG^ particles were shorter, some of them only 500 nm, and thinner than wild type reference particles (Fig 7). The isolated PVA^WT^ and PVA^AG^ particles are provided in Fig 7 as size references to which the particle stacks can be compared. The length of the isolated PVA^WT^ particle was estimated to be around 730 nm and that of PVA^AG^ around 500 nm. Based on these results some particles were formed in PVA^AG^ infected cells but they appeared misassembled and partially degraded. S4 Fig demonstrates that viral gene expression level was equal in the samples, although the amounts of particles in the respective tissues were drastically different.

### Co-localization and co-immunopurification revealed an HCPro-mediated association between AGO1 and CP

**Fig 8:**
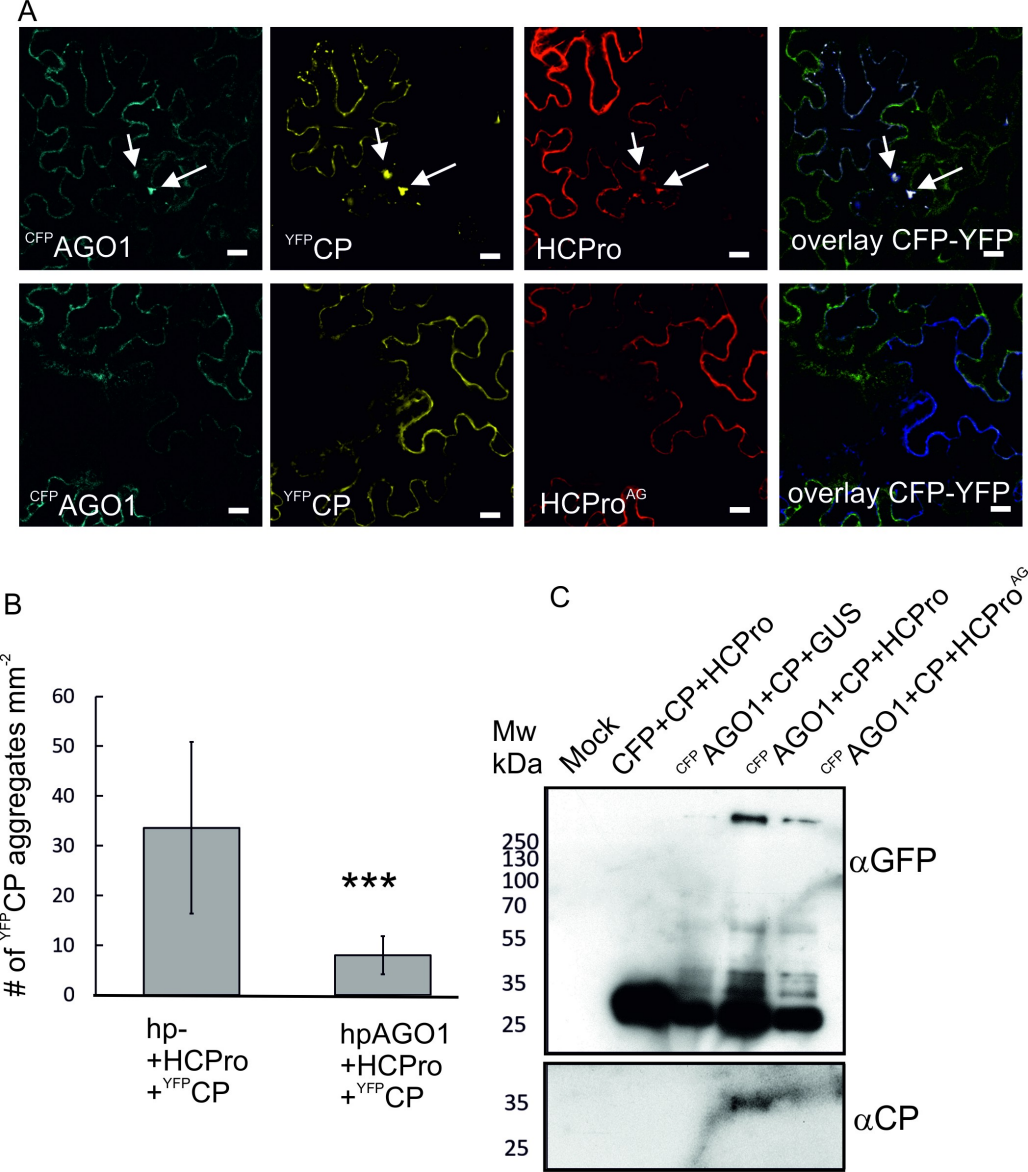
HCPro mediates the association between AGO1 and CP. A) ^YFP^CP and ^CFP^AGO1 co-localize in cytoplasmic foci in the presence of HCPro but not in the presence of HCPro^AG^. ^YFP^CP, ^CFP^AGO1 and HCPro/HCPro^AG^ were co-expressed transiently at OD_600_ 0.1. Infiltrated leaves were imaged by confocal microscopy at 3 dpi. For control images showing unfused YFP, CFP and RFP as controls for ^YFP^CP, ^CFP^AGO1 and HCPro/HCPro^AG^, respectively, see S3 Fig. Scale bar 20 μm. B) The silencing of *AGO1* significantly reduced the number of cells displaying ^YFP^CP foci. The silencing constructs were infiltrated at OD_600_ 0.4 while overexpression constructs were infiltrated at OD_600_ 0.1. Leaf disks were collected at 3 dpi and epifluorescence microscopy was used to count the number of epidermal cells per mm^2^ containing ^YFP^CP aggregates. Results are averages of three independent experiments, error bars denote standard deviation (*** P<0.001). C) CP co-immunoprecipitates with ^CFP^AGO1 depending on the presence of HCPro. ^CFP^AGO1 and CP were co-infiltrated with HCPro/HCPro^AG^ or GUS as control. Samples were collected at 3 dpi. GFP trap was used for affinity purification followed by western blot analysis of the eluates to detect ^CFP^AGO1 (top panel) and associated CP (bottom panel). Validation of CP expression in the input is presented in S5 Fig.

Because CP was more stable in the presence of HCPro than HCPro^AG^, we decided to study if the intracellular localization of YFP-tagged CP was affected by AGO1, HCPro/HCPro^AG^ or their combinations. When all three proteins, ^YFP^CP, ^CFP^AGO1 and RFP-tagged HCPro were co-expressed and leaf samples were inspected under a confocal microscope, we observed that ^YFP^CP and ^CFP^AGO1 co-localized strongly in distinct cytoplasmic aggregates (Fig 8A). These aggregations of ^YFP^CP and ^CFP^AGO1 were not observed when HCPro was replaced by HCPro^AG^. In fact, their formation was entirely dependent on HCPro as we did not observe any ^YFP^CP/^CFP^AGO1 aggregations during co-expression with the RFP control (S3C Fig). YFP alone did not form distinct aggregations but was evenly spread in the cytoplasm and nucleus (S3C Fig).

In the presence of HCPro^AG^ both ^CFP^AGO1 and ^YFP^CP were more uniformly distributed throughout the cytoplasm. Analysis of controls revealed that ^YFP^CP forms distinct, although smaller, spots also when co-expressed with the CFP control and HCPro suggesting that the level of endogenous AGO1 could be enough to induce the sequestration of ^YFP^CP in response to HCPro. In line with this, silencing of AGO1 by a hairpin construct significantly decreased the number of cells containing ^YFP^CP foci (Fig 8B).

Some viral CPs are known to interact with AGO proteins through WG/GW motifs [53, 59]. According to a sequence search, the PVA CP did not contain any putative AGO1 binding motifs. Nevertheless, we used affinity purification to assess if CP could be co-purified with AGO1 and found that CP and AGO1 indeed co-immunoprecipitate, but only in the presence of HCPro (Fig 8C). In these experiments CP and HCPro, HCPro^AG^ or GUS were transiently co-expressed with either ^CFP^AGO1 as bait or CFP as negative control. Validation of CP expression in the inputs is presented in S5 Fig. Consistent to [34], accumulation of CP is prominent only when HCPro is co-expressed. Samples were obtained at 3 dpi and a GFP trap was used to affinity purify ^CFP^AGO1 and associated protein partners. Western blotting with anti-GFP and anti-CP antibodies was used to detect proteins in the eluates. ^CFP^AGO1 was mainly present in a high molecular weight complex corresponding to results from strep-tag purifications (Figs 4B and 8C). A strong band at circa 27 kDa suggests ^CFP^AGO1 is proteolytically processed to some extent and that the CFP fusion partner can be detached from AGO1. A CP signal was detected when its expression was combined with ^CFP^AGO1 and HCPro. No CP could be detected in the controls lacking either ^CFP^AGO1 or HCPro (Fig 8C). Overall the co-immunopurification results correlated well with the microscopy results although in one biological repeat very little CP co-immunoprecipitated also when HCPro^AG^ was used instead of the wild type protein. Thus it is possible that while HCPro’s ability to mediate the association between CP and AGO1 was weakened by the W208A mutation it was not completely disabled.

## Discussion

We report here a unique pro-viral role for plant AGO1 in the stability and accumulation of potyvirus particles. In addition, we demonstrate that the viral HCPro and, more specifically, a conserved WG motif therein, is responsible for engaging AGO1 in the promotion of the infection. When the virus lacked HCPro completely, it fell prey to AGO1’s defensive activities. This was observed as a decrease in vRNA level responsive to AGO1 overexpression and a corresponding increase while *AGO1* was silenced (Fig 1G and 1H). The generation and characterization of a novel HCPro mutant with impaired ability to associate with AGO1 underlined the biological importance of the interaction for virus particle yield. We found that a fully functional connection between HCPro and AGO1 was required for stable encapsidation and, hence, for optimal systemic spread of potyviruses. In support, our results indicated that the viral CP co-localized and co-immunoprecipitated with ^CFP^AGO1 only in the presence of HCPro and was more vulnerable to degradation if the HCPro-AGO1 interaction was compromised. We show that CP’s increased vulnerability is due to a destabilization of virus particles linked to the mutation in HCPro. Thus, taken together, we propose that the potyviral HCPro recruits AGO1 through its WG motif and employs it in the assembly of stable virus particles.

Most of the viral silencing suppressors interact with AGO1 via a specific motif called WG/GW. The motif derives its name from characteristic tryptophan (W) -glycine (G) residue pairs and specifically binds the PIWI domain that conveys slicer activity to AGO proteins [63–66]. Sequence searches revealed a putative AGO-binding WG motif in PVA HCPro and multiple alignment of 113 potyvirus polyproteins indicated the motif was conserved in almost all of the HCPro sequences (Fig 2, S1 Table), BBrMV making the sole exception (S2 Fig). Based on our results, HCPro’s silencing suppressor activity appears to be independent of its ability to interact with AGO1.

The potyviral HCPro has been shown to sequester sRNAs of viral origin [42, 67]. Moreover, *Arabidopsis* AGO1 hosted TuMV-derived vsiRNAs only in the presence of a silencing suppression deficient HCPro [42]. The potyviral HCPro can be divided into three regions: the N-terminal region involved in aphid transmission (amino acids 1–100 in PVA HCPro), the functionally rich central region (amino acids 100–300), and the C-terminal serine proteinase region (amino acids 300–457; [68]). The central region contains domains for RNA- and siRNA-binding [68–70] and it is involved in RNA silencing suppression [71]. Essential functional properties such as systemic movement [36, 72], synergistic interaction with other viruses [73, 74] and virion formation [34] have been associated with this domain as well. To investigate the possibility of secondary effects of the W208A mutation located in the central region, HCPro^AG^ was subjected to a number of assays designed to test its functionality. It successfully complemented viral gene expression level of the HCPro-less virus, retained silencing suppression capacity in our dsRNA hairpin test and enhanced translation normally in collaboration with VPg. These results emphasize that HCPro^AG^ maintained central functional properties required for stages preceding encapsidation. Structural models of both HCPro^WT^ and HCPro^AG^ were prepared using I-TASSER server for protein structure predictions. The overlay of the predicted structures indicates that HCPro’s overall structure along with the known functional motifs were not disturbed due to the W208A mutation (S6 Fig). Taken together, the functional data and the structural model suggest that the W208A mutation disrupts the AGO1 binding site locally without altering other functions of HCPro.

A tobacco etch virus (TEV) mutant called PC22, which has an asparagine changed to serine in the immediate vicinity of the WG-motif of HCPro (**N**GNFI**WG**LR→**S**GNFI**WG**LR), did not accumulate in systemically infected plants as efficiently as the wild type virus in a competition assay [75]. Nevertheless the HCPro of PC22 had an equally efficient silencing suppression activity as the wild type HCPro [76]. Interestingly, another mutation (RRH234-236AAA) in PPV HCPro located close to the WG motif affected CP stability, but did not affect its RNA-silencing suppression activity [34]. This mutation had an effect on PPV particle formation. Taken together, accumulated evidence strongly suggests the central region of potyviral HCPro close to the AGO1-binding motif is involved in long-distance movement and particle formation. While it is not possible to conclusively extrapolate the role of AGO1-HCPro interaction in PPV and TEV infections, we propose that it is central for these phenomenon in the cases of PVA and TuMV.

The AGO family of RNA silencing effectors holds an established position in plant antiviral defense (reviewed in [8, 21]). AGO1 was thought to be the major player in antiviral activities until an investigation of turnip crinkle virus and cucumber mosaic virus infections in mutant *Arabidopsis* plants revealed that AGO2 provides an important secondary layer of defense [40]. A study of TuMV infection in *Arabidopsis* revealed AGO1 contributed to antiviral defense mainly in systemically infected inflorescence tissues where its effects blended in with AGO10 activity [42]. Later, in [60], the authors inactivated *N*. *benthamiana AGO2* by Crispr/Cas9 -mutagenesis and showed its importance in defense against potato virus X, TuMV and turnip crinkle virus. In a healthy plant AGO1 is prevalent and downregulates AGO2 expression via miR403 [61]. During an infection, when AGO1 is targeted by viral silencing suppressors, AGO2 expression is upregulated. This consequently increases its impact on host antiviral defense [40]. Interestingly, data obtained from the *Arabidopsis*-cucumber mosaic virus pathosystem implies AGO1 and AGO2 can also engage in non-redundant co-operation against the virus by specializing in distinct types of vsiRNAs [62].

The interdependent relationship between AGO1 and AGO2 is relevant for a balanced defense response. Thus, we measured the changes in *AGO2* mRNA levels in our *AGO1* silencing experiments. We found that indeed the expression of *AGO2* mRNA was enhanced in *AGO1*-silenced samples (S1 Fig). It is possible that this results in a shift in the AGO1/AGO2 balance leading to the unmasking of AGO2-dependent defense during PVA infection. From this point of view the pro-viral effect of AGO1 would be indirect because the higher AGO2 levels could explain the negative impact the silencing of *AGO1* had on the infection. Although virus-derived gene expression did not decrease significantly during *AGO1* silencing, we acknowledge the possible additional effects of increased AGO2 activity in these circumstances.

An important viral strategy to subdue the host’s silencing machinery is to encode proteins with silencing suppression activity. Apparently, due to its central position in the RNA silencing mechanism AGO1 has emerged as a major target for viral silencing suppressors. Data gathered from across plant virus families reveals that a wide range of silencing suppressor proteins interfere with host AGO1. For example, the serine proteinase P1 protein of sweet potato mild mottle virus (SPMMV; genus *Ipomovirus*; family *Potyviridae*) binds AGO1 via a WG-motif [49]. Instead of HCPro, P1 serves as the RNA silencing suppressor in SPMMV infection [49]. SPMMV HCPro does not contain either the AGO1-binding WG motif or the typical potyviral consensus sequence around it. P1N-PISPO protein encoded by sweet potato feathery mottle virus (SPFMV; genus *Potyvirus*) contains a WG-motif. The WG motif of PISPO is required for its silencing suppression activity [77], but it is not known if it is able to bind to AGO. On the other hand, SPFMV HCPro has retained the typical potyviral WG-motif consensus sequence. This proposes that the functions of AGO1-HCPro binding in infection in the close relatives of potyviruses, the ipomoviruses, may have been substituted with some alternative mechanisms. Protein p25 of turnip crinkle virus (family *Tombusviridae*) as well as 2b of cucumber mosaic virus (family *Bromoviridae*) bind AGO1 in order to inhibit its activity [44, 47, 49]. Interaction between the P0 protein of beet western yellows virus and cucurbit aphid-borne yellows virus (family *Luteoviridae*) and host AGO1 leads to AGO1 degradation via autophagy [45, 46, 50, 51]. Likewise potato virus X (family *Alphaflexiviridae*) p25 and enamovirus (family *Luteoviridae*) P0 have been suggested to alleviate defensive pressure on the infection by directing AGO1 to degradation [48, 52]. We used confocal microscopy-based co-localization studies and affinity purification to show that the potyviral HCPro indeed associates with AGO1. As the association was significantly reduced by the W208A mutation, we propose the two proteins associate with each other *in vivo*.

In the current study the systemic spread of two potyviruses, PVA and TuMV, was significantly reduced when the W208A mutation was introduced to the conserved WG motif in HCPro. The effect of the mutation on virus particles was more drastic as it nearly abolished the accumulation of virions (Fig 6). We demonstrate the mutation was detrimental to not only the interaction between PVA HCPro and AGO1 but also for enabling the association between AGO1 and CP. We interpret that poor CP stability in a PVA^AG^ infection is indicative of its degradation due to reduced incorporation into particles or instability of the assembled particles. Based on this evidence we suggest that AGO1 makes positive contributions to potyvirus encapsidation under HCPro’s coordination and that it has a key role in ensuring the stability of the particles.

The ability to separate AGO1-binding functions from other biochemical properties of the viral silencing suppressor is not a unique feature of HCPro: for example, AGO1-interaction of the cucumber mosaic virus 2b protein has also been shown to be independent of its silencing suppression activity [78]. Likewise, the silencing suppressor activity of the pelargonium line pattern virus (family *Tombusviridae*) coat protein p37 was independent of its ability to interact with AGO1 and AGO4 in *N*. *benthamiana* [59]. According to the study p37’s ability to suppress silencing depended foremost on its sRNA-binding capacity. Our data supports the scenario where the lack of HCPro could result in vsiRNA enrichment within AGO1, which consequently targets vRNA as a slicer. Because the HCPro^AG^ mutant retained its capacity to suppress silencing we expect it also sequesters vsiRNAs normally.

Particle production is a crucial step for the spread of the virus as encapsidation ensures the viral genome is protected from degradation. For many plant viruses, particles are the major form of long-distance movement (reviewed in [58]). For potyviruses virions might not be the only option for systemic movement as replication vesicles have been discovered both in the phloem and xylem [79–81]. We found that despite extremely low particle amounts detected by IC-RT-PCR and EM both PVA^AG^ and TuMV^AG^ were able to establish systemic infections, albeit with diminished efficiencies (Figs 5 and 6). Our results gave rise to two possibilities: either particles were not formed or the assembled particles were so unstable they were degraded immediately after cell lysis required by IC-RT-PCR and EM analyzes. In the former case, systemic spread could be explained by long-distance transport of vRNA in replication vesicles or as part of ribonucleoprotein complexes instead of as virus particles. The electron micrographs of the infected leaf tissues provided more evidence for the latter option. EM of thin sections of infected leaves showed that PVA^AG^ produced particles which, however, appeared malformed and undersized. We conclude that the lack of the stabilizing effect of CP-AGO1-HCPro interaction makes the particles sensitive to cellular proteases and RNases, leading to disassembly of particles and degradation of CP. We propose that in spite of being distorted the stability of the both PVA^AG^ and TuMV^AG^ particles was enough to support long-distance movement in intact plants. However, the reduced efficiency of long-distance movement may reflect that less PVA^AG^ and TuMV^AG^ particles succeeded in protecting PVA RNA during phloem transport than PVA^WT^ or TuMV^WT^ particles.

The mechanism of potyviral encapsidation and the required components are not yet fully understood. Although the signal for and origin of encapsidation remain obscure, and may rely on protein-protein interactions rather than protein-RNA interaction [82], Gallo et al. [35] recently demonstrated that only replication-competent vRNA is encapsidated. Successful encapsidation of PPV requires CP stabilization by HCPro [34]. Recent research has also proposed an active role for CP in sequestering vRNA from translation to encapsidation [82]. Torrance et al. [83] used atomic force microscopy to study the detailed architecture of potyvirus particles. The high-resolution technique revealed that mature virions can contain a tip structure located at one end. In other plant viruses, such protein complexes have been linked to enhanced particle stability or controlled disassembly [84–86]. In PVA and potato virus Y the structures contained HCPro and VPg [83]. The authors proposed the complex forms at the 5’ UTR of the vRNA because VPg’s is covalently attached to this position. Also the CI, a viral helicase, was later found to associate with one end of potyvirus particles indicating that it could form part of the structure [87]. We have earlier sketched the concept of a virus-induced complex involving HCPro and AGO1 and proposed it could function in relieving translational repression [54]. Based on results presented here, we suggest HCPro not only attracts AGO1 to the complex on vRNA but also serves as a bridge between AGO1 and CP. We hypothesize that the interaction follows vRNA from translation to encapsidation. When correctly assembled the interface between CP and the RNP-complex or the “tip”-structure as in [83, 87] needs to be sealed stably and HCPro-AGO1-CP interaction may be crucial in this function. When HCPro’s capacity to interact with AGO1 was compromised by the W208A mutation, consequences for the infection were detrimental. We suggest that in PVA^AG^ a weakened interaction between HCPro, AGO1 and CP could produce fragile particles with incomplete tip structures that lack the stabilizing effect of AGO1. The inability of PVA^AG^ and TuMV^AG^ to accumulate normal amounts of particles could be a consequence of this instability. Similar circumstances could occur during the silencing of *AGO1* when host AGO1 levels are too low to support stable encapsidation.

In summary, potyviral HCPro’s capacity to interact with AGO1 agreed with previous findings linking viral silencing suppressors to AGO proteins. WG/GW motif mimicry among viral silencing suppressors can be advantageous in directing host AGO proteins away from their antiviral functions. Our results, however, point to the direction that HCPro not only tames AGO1’s aggressive antiviral activity but also employs it in pro-viral functions such as virus particle stabilization. The high conservation of the AGO1 binding site in HCPro suggests strong evolutionary pressure to maintain this site. The need for stable particles for aphid transmission may act as a driving force here. The finding is an interesting example highlighting the evolutionary arms race between viruses and their hosts. We anticipate that the emerging role for AGO1 in potyvirus infections calls for further studies in order to clarify the ongoing coevolution of plant defense and viral counter defense.

## Methods

### Plants and growth conditions

*Nicotiana benthamiana* plants were grown in a greenhouse environment under a 16 h light and 8 h dark cycle at 22 ^o^C and 18 ^o^C respectively with a relative humidity of circa 50%. Plants were used in the experiments at the four-to-six-leaf stage.

### Construction of recombinant viruses

PVA constructs used in this study are based on the PVA-B11 full-length infectious cDNA (GenBank accession no. AJ296311) expressed under the cauliflower mosaic virus 35S promoter and tagged with the *Renilla* luciferase reporter containing a plant intron. To enable *Agrobacterium*-mediated expression in plants all virus constructs were incorporated into the pBIN19 binary vector (GenBank: U09365.1). PVA^WT^ and the replication-deficient PVA^ΔGDD^ were described in [55] while the HCPro-less viruses PVA^ΔHCPro^ and PVA^ΔGDDΔHCPro^ were described in [39].

The Agos computational tool, available online at http://www.combio.pl/agos/, was used to detect putative ARGONAUTE-binding sites in the PVA HCPro sequence [56, 57]. The W208A mutation was introduced into the HCPro of PVA using the site-directed mutagenesis method described in [88]. A pGEM-T Easy vector (Promega) carrying the SexAI-NruI -fragment (contains the P1 C-terminal, HCPro, P3 N-terminal) from the PVA icDNA was used as the template in two separate PCR reactions, one with the forward primer 5’-CAACTTGATA**GCG**GGCGAGAGAG -3´and the other with the reverse primer 5´-CTCTCTCGCC**CGC**TATCAAGTTG-3´. The mutated codon is designated in bold. The Phusion high fidelity polymerase (Thermo Fisher Scientific) was used for all PCR-based cloning steps.

The presence of the W208A mutation was verified by sequencing and the SexAI-NruI fragment was then used to replace the wild type fragment in PVA icDNA.

TuMV constructs are based on the UK1 strain icDNA expressed under the cauliflower mosaic virus 35S promoter in the pCB301 binary vector backbone (GenBank accession no: EF028235.1). The recombinant TuMV construct carrying the GFP reporter between P1 and HCPro has been described in [89]. Additionally the expression plasmid contains the mCherry reporter gene under an independent 35S promoter within the bordering T-DNA sequences [90].

The mutation was introduced into TuMV icDNA by a similar method as for the corresponding mutation in PVA. First the StuI-SnaBI fragment was PCR amplified from the TuMV icDNA in p35-Tunos-Vec0-NAT1-GFP [91] with the primers 5´-CAGAAACGAAGCAGAAGTC-3´and 5´-CTTGCTACTAGTCCAAGACATAC-3´ and cloned into pGEM-T Easy (Promega). For site-directed mutagenesis the forward and reverse PCR reactions were primed with 5´-AATTTCATA**GCG**GGTGAGAGAG-3´ and 5´-TCTCTCACC**CGC**TATGAAATTTC-3´, respectively. The mutation was transferred to TuMV icDNA in the pCB301-TuMVGFP//mcherry plasmid by overlapping extension PCR combining the GFP reporter and HCPro. The GFP-HCPro^AG^ fragment was cloned into pGEM-T Easy (Promega) followed by AgeI-Bsu36I restriction and ligation into similarly double-digested pCB301-TuMVGFP//mCherry.

### Overexpression constructs

To generate a transient overexpression construct of TwinStrep-tagged RFP fusion of the PVA HCPro^AG^ the W208A mutation was introduced into a P1-TwinStrep-RFP-HCPro-P3 fragment in pGEM-T Easy (Promega) with site-directed mutagenesis using the oligos described above. The sequence was then PCR amplified with oligos adding an XhoI restriction site and a start codon to the 5´ end and a stop codon and a BamHI restriction site to the 3´ end (Forward oligo 5´-TCCGCTCGAGATGAAATGGTCTCATCCACAA-3´and reverse 5´-CGCGGATCCTCATCCAACCCTGTAGTGCT-3´). The PCR product was digested with XhoI and BamHI and ligated into an XhoI-BamHI double-digested interim vector (pHTT690) to provide the 35S promoter and *nos* terminator. After sequence verification the 35S-TwinStrep-RFP-HCPro^AG^-nos expression cassette was transferred into the pBIN19 binary vector using HindIII restriction sites.

To generate a YFP-tagged CP, PVA CP gene was amplified from the PVA icDNA with primers incorporating Gateway-compatible attB sequences. The PCR product was cloned into pDONRZeo and finally into destination vectors using Gateway BP and LR reactions, respectively (Thermo Fisher Scientific). pGWB42 was used as the destination vector to generate an N-terminal fusion of the CP. The pGWB series binary expression vectors have been described in [92]. The ^CFP^AGO1 expression construct was as in [39].

### Agroinfiltration

All constructs were delivered into Agrobacteria by electroporation. *Agrobacterium* strain C58C1 was used as the host bacterium for introducing PVA, protein overexpression and silencing constructs into *N*. *benthamiana* plants. *Agrobacterium* strain GV3101 (pMP90) was used as the host bacterium for TuMV constructs. *Agrobacteria* harboring the desired plasmids infiltrated into *N*. *benthamiana* leaves as described previously [54].

### Dual luciferase assay

The dual luciferase assay was performed with the Dual-Luciferase Reporter Assay System (Promega E1910/E1960) as in [55].

### SDS-PAGE and western blotting

The separation of proteins on SDS-PAGE gels and western blotting were done according to standard procedures. HCPro and CP were detected with polyclonal antibodies produced in rabbit. An HRP-conjugated anti-rabbit antibody was used as the secondary antibody (Promega; W4011). CFP and YFP and their fusions were detected with a mouse monoclonal GFP antibody (Santa Cruz; SC-9996) and RFP with a mouse monoclonal RFP antibody (SignalChem; R46-61M-100). RLUC was detected with a mouse monoclonal antibody (Millipore; MAB4400). The secondary antibody for the monoclonal antibodies was an HRP-conjugated anti-mouse antibody (Promega; W4021).

### RNA extraction and qRT-PCR

For gene expression analysis total RNA was extracted from 100 mg leaf samples with the Gene Jet Plant RNA Purification kit (Thermo Fisher Scientific) according to the manufacturer’s instructions. Prior to cDNA synthesis contaminating T-DNA molecules carrying PVA cDNA and genomic DNA carrying the PP2A and F-box reference genes were removed with RQ1 RNase free DNAseI (Promega) treatment at 37 ^o^C for 30 min. cDNA was synthesized with the RevertAid H Minus First Strand cDNA Synthesis kit (Thermo Fisher Scientific) as recommended by the manufacturer using random hexamers as primers. Quantitative real-time PCR was done using the Maxima SYBR Green qPCR Master Mix (Thermo Scientific). PVA CP was amplified with 5´-CATGCCCAGGTATGGTCTTC-3´and 5´-ATCGGAGTGGTTGCAGTGAT-3´ and TuMV CP with 5´-TGGCTGATTACGAACTGACG-3´and 5´-CTGCCTAAATGTGGGTTTGG-3´. *N*. *benthamiana* PP2A and Fbox were used as reference genes [14] and were amplified with 5´-GACCCTGATGTTGATGTTCG-3´and 5´-GAGGGATTTGAAGAGAGATTTC-3´ (PP2A) and 5´-GGCACTCACAAACGTCTATTTC-3´and 5´-TGGGAGGCATCCTGCTTAT-3´ (Fbox). Results were normalized to reference gene expression with the ΔΔCq method.

### Immunocapture of virions and RT-PCR

For IC RT-PCR tubes were coated with the anti-CP antibody in ELISA coating buffer for 3 h at 37 ^o^C followed by washing with ELISA washing buffer. 100 mg leaf samples were homogenized, resuspended in ELISA sample extraction buffer and allowed to settle on ice for 1 h. Samples were incubated in the CP-antibody-coated tubes overnight at 4°C. After incubation the tubes were washed twice with ELISA washing buffer, once with 1 × PBS and once with distilled water. cDNA synthesis was primed with random hexamers and the tubes were heated for 5 min at 65 ^o^C to disrupt bound virions. The rest of the components of the RevertAid H minus First Strand cDNA synthesis kit (Thermo Fisher Scientific) were then added and cDNA was synthesized according to the manufacturer’s protocol. The cDNA was used as template in qPCR to determine vRNA copy number originating from captured particles. Copy numbers were calculated based on a linear standard generated from serial dilutions of a plasmid template in the same qPCR run.

### Affinity purification and co-immunoprecipitation

HCPro/HCPro^AG^ and interacting partners were immunoprecipitated by affinity purification adapted from the method described in [93].

*N*. *benthamiana* plants were infiltrated with *Agrobacteria* carrying HCPro or HCPro^AG^ as bait plus ^CFP^AGO1 as target or CFP as a negative control. Each overexpression construct was infiltrated at OD_600_ 0.1 and samples were collected at three dpi. 1 g of frozen leaf material was ground in liquid nitrogen and resuspended in 3 ml of binding buffer (25 mM Tris-HCl pH 8.0, 550 mM NaCl, 5 mM NaF, 0.5 mM EDTA, 10% Glycerol, 0.1 mM PMSF supplemented with Pierce protease inhibitor tablets). Samples were then cleared by filtering through double-layered Miracloth followed by a 5 min centrifugation at 5000 × g at 4°C. To minimize the binding of endogenous biotinylated proteins to the StrepTactin resin, samples were supplemented with 100 μg/ml avidin and incubated for 15 min at 4 ^o^C in rotation. The cross-linked StrepTactin was mixed by vortexing before adding 50 μl of resin per sample. Samples were rotated for 2 h at 4 ^o^C to allow binding of the bait proteins to the resin. Recovered resin was washed thrice in wash buffer #1 (25 mM Tris-HCl pH 8.0, 500 mM NaCl, 5 mM NaF, 0.4 mM EDTA, 0.2% Igepal CA-630, 5% Glycerol, 0.1 mM PMSF) and once in buffer #2 (25 mM Tris-HCl pH 8.0, 150 mM NaCl) using spin column tubes (Costar Spin-X 0.45 um cellulose). The samples were eluted in 25 mM Tris-HCl pH 8, 1% SDS buffer for 5 min at 55 ^o^C and 1400 rpm shaking. The bait protein and co-immunoprecipitated targets were detected from the eluates by SDS PAGE followed by western blotting. ^CFP^AGO1 was affinity purified similarly by using a GFP-trap (Chromotek) according to the manufacturer’s instructions.

### Confocal and epifluorescence microscopy

Viral and host proteins tagged with fluorescent markers were transiently expressed in *N*. *benthamiana*. All constructs were agroinfiltrated at an OD_600_ of 0.1 and infiltrated leaves were examined at 3 dpi. The localization of fluorescent fusion proteins was studied with confocal laser scanning microscopy using a Leica TCS SP5II or SP8 instrument (Leica Microsystems). All images were acquired with a 63x water immersion objective and in sequential scanning mode in order to avoid fluorophore bleed-through. YFP and RFP were excited with a 488 nm Argon laser and emission was detected at 522–552 nm and at 566–618 nm, respectively. CFP was excited with a 405 nm laser and emission was detected at 461–502 nm. Leaf samples were mounted upside down on an objective slide with a drop of water and placed under a cover glass.

Co-localization analysis of ^CFP^AGO1 with HCPro and HCPro^AG^ was performed with the ImageJ image analysis software. HCPro-containing granules were selected as ROIs and co-localization threshold function was used to calculate the amount of co-localized pixels from the RFP and CFP channels. Results are given as % of HCPro/HCPro^AG^ co-localizing with ^CFP^AGO1.

Epifluorescence microscopy of *N*. *benthamiana* epidermal cells was done with a Zeiss Axioskop2 plus instrument using a FITC filter to detect YFP fluorescence.

### Transmission electron microscopy

Virus particles were visualized by negative staining followed by transmission electron microscopy with a Jeol JEM-1400 instrument (Jeol Ltd., Tokyo, Japan). 100 mg leaf samples were homogenized and suspended in cold 0.06 M phosphate buffer. While letting the samples clear on ice, carbon-coated EM-grids were coated for 1 h at RT with an anti-CP antibody (PVA) at 1:100 dilution. The grids were then washed once with phosphate buffer and incubated for 5 min with cleared sample lysate. After washing with 20 drops of phosphate buffer the grids were stained for 15 s with 2% uranyl acetate. EM images of the infected leaf tissues were visualized following Lõhmus et al. [94].

## Supporting information

S1 Fig Validation of silencing and overexpression of AGO1 for the experiments shown in Fig 1. (A) Validation of the AGO1 silencing in mRNA level in Fig 1A–1C. Demonstrated is also upregulation in Ago2 mRNA due to AGO1 silencing. (B) AGO1-CFP over-expression corresponding to Fig 1D–1F verified by α-CFP western blot. Ponceau-S staining of RuBisCo shows equivalent loading (C) Validation of AGO1 silencing (upper panel) and over-expression (lower panel), related to Fig 1G and 1H, by RT-PCR at mRNA level. The mRNA levels of the housekeeping gene PP2A served as a loading control.
(TIF)

S2 Fig Alignment of banana bract mosaic virus (BBrMV) and PVA HCPro. A comparison of HCPro sequences of seven BBrMV isolates with PVA HCPro revealed the absence of WG motif in BBrMV. ‘W’ is in the same position as in other potyviruses but ‘G’ is substituted by ‘N’ in this virus.
(TIF)

S3 Fig Control images for confocal microscopy experiments. A) Control studies for co-localization of HCPro and HCPro^AG^ together with ^CFP^AGO1 in *N. benthamiana* leaves. Unfused RFP was used as a control for HCPro/HCPro^AG^ and CFP for ^CFP^AGO1. All constructs were agroinfiltrated at OD_600_ 0.1 and leaves were imaged by confocal microscopy at 3 dpi. B) Validation of the AGO1^CFP^ expression in mRNA level in Fig 4B overexpression C) Control studies for co-localization of ^YFP^CP together with ^CFP^AGO1 and HCPro/HCPro^AG^. Unfused RFP was used as a control for HCPro/HCPro^AG^, YFP for ^YFP^CP and CFP for ^CFP^AGO1. Representative images from three independent experiments are shown, scale bar is 20 μm.
(TIF)

S4 Fig Validation of equivalent expression of PVA^WT^ and PVA^AG^ presented in Fig 7. PVA^WT^ and PVA^AG^ expression level determined by the RLUC assay (upper panel) and particle amounts determined by IC-RT-PCR (lower panel) corresponding to the EM images shown in Fig 7.
(TIF)

S5 Fig Validation CP expression in the input for sets presented in Fig 8C. (TIF)

S6 Fig Overlay of PVA HCPro^WT^ and HCPro^AG^ structures. Structural predictions of HCPro^WT^ (in white) and HCPro^AG^ (in blue) were independently made using I-TASSER protein prediction tool and server. Models consistent to TuMV HCPro crystal structure (PDB id-3RNV) were overlaid using Chimera protein structure visualization software. Both the structures matched well and positions of several important functional motifs of HCPro (in yellow) were intact. Finally, WG (in green) and its mutated counterpart AG (in red) were also found to be in close vicinity, however, their molecular structure varied as amino acid ‘W’ was replaced with ‘A’ (in the box).
(TIF)

S1 Table Conservation of the WG motif in 113 potyviral HCPros. Related to Fig 2.
(DOCX)

S1 Data Supplemental data file which provides all numerical values used for drawing the graphs. (XLSX)

## References

[1] Scholthof KB, Adkins S, Czosnek H, Palukaitis P, Jacquot E, Hohn T, et al Top 10 plant viruses in molecular plant pathology. Mol Plant Pathol. 2011;12(9):938–54. Epub 2011/10/21. 10.1111/j.1364-3703.2011.00752.x 22017770 PMC6640423

[2] Sanfaçon H. Grand Challenge in Plant Virology: Understanding the Impact of Plant Viruses in Model Plants, in Agricultural Crops, and in Complex Ecosystems. Front Microbiol. 2017;8:860 Epub 2017/05/24. 10.3389/fmicb.2017.00860 28596756 PMC5442230

[3] Yang Z, Li Y. Dissection of RNAi-based antiviral immunity in plants. Curr Opin Virol. 2018;32:88–99. Epub 2018/10/31. 10.1016/j.coviro.2018.08.003 . 30388659

[4] Zhang W, Zhao F, Jiang L, Chen C, Wu L, Liu Z. Different Pathogen Defense Strategies in. Cells. 2018;7(12). Epub 2018/12/07. 10.3390/cells7120252 30544557 PMC6315839

[5] Baulcombe DC. VIGS, HIGS and FIGS: small RNA silencing in the interactions of viruses or filamentous organisms with their plant hosts. Curr Opin Plant Biol. 2015;26:141–6. Epub 2015/08/03. 10.1016/j.pbi.2015.06.007 . 26247121

[6] Guo Z, Li Y, Ding SW. Small RNA-based antimicrobial immunity. Nat Rev Immunol. 2019;19(1):31–44. 10.1038/s41577-018-0071-x . 30301972

[7] Li F, Wang A. RNA-Targeted Antiviral Immunity: More Than Just RNA Silencing. Trends Microbiol. 2019;27(9):792–805. Epub 2019/06/15. 10.1016/j.tim.2019.05.007 . 31213342

[8] Fang X, Qi Y. RNAi in Plants: An Argonaute-Centered View. Plant Cell. 2016;28(2):272–85. Epub 2016/02/11. 10.1105/tpc.15.00920 26869699 PMC4790879

[9] Bohmert K, Camus I, Bellini C, Bouchez D, Caboche M, Benning C. AGO1 defines a novel locus of Arabidopsis controlling leaf development. EMBO J. 1998;17(1):170–80. 10.1093/emboj/17.1.170 9427751 PMC1170368

[10] Höck J, Meister G. The Argonaute protein family. Genome Biol. 2008;9(2):210 Epub 2008/02/26. 10.1186/gb-2008-9-2-210 18304383 PMC2374724

[11] Zheng X, Zhu J, Kapoor A, Zhu JK. Role of Arabidopsis AGO6 in siRNA accumulation, DNA methylation and transcriptional gene silencing. EMBO J. 2007;26(6):1691–701. Epub 2007/03/01. 10.1038/sj.emboj.7601603 17332757 PMC1829372

[12] Havecker ER, Wallbridge LM, Hardcastle TJ, Bush MS, Kelly KA, Dunn RM, et al The Arabidopsis RNA-directed DNA methylation argonautes functionally diverge based on their expression and interaction with target loci. Plant Cell. 2010;22(2):321–34. Epub 2010/02/19. 10.1105/tpc.109.072199 20173091 PMC2845420

[13] Wei W, Ba Z, Gao M, Wu Y, Ma Y, Amiard S, et al A role for small RNAs in DNA double-strand break repair. Cell. 2012;149(1):101–12. Epub 2012/03/22. 10.1016/j.cell.2012.03.002 . 22445173

[14] Liu D, Shi L, Han C, Yu J, Li D, Zhang Y. Validation of reference genes for gene expression studies in virus-infected Nicotiana benthamiana using quantitative real-time PCR. PLoS One. 2012;7(9):e46451 Epub 2012/09/28. 10.1371/journal.pone.0046451 23029521 PMC3460881

[15] Takeda A, Iwasaki S, Watanabe T, Utsumi M, Watanabe Y. The mechanism selecting the guide strand from small RNA duplexes is different among argonaute proteins. Plant Cell Physiol. 2008;49(4):493–500. Epub 2008/03/14. 10.1093/pcp/pcn043 . 18344228

[16] Mi S, Cai T, Hu Y, Chen Y, Hodges E, Ni F, et al Sorting of small RNAs into Arabidopsis argonaute complexes is directed by the 5' terminal nucleotide. Cell. 2008;133(1):116–27. Epub 2008/03/13. 10.1016/j.cell.2008.02.034 18342361 PMC2981139

[17] Vaucheret H. Plant ARGONAUTES. Trends Plant Sci. 2008;13(7):350–8. Epub 2008/05/26. 10.1016/j.tplants.2008.04.007 . 18508405

[18] Fagard M, Boutet S, Morel JB, Bellini C, Vaucheret H. AGO1, QDE-2, and RDE-1 are related proteins required for post-transcriptional gene silencing in plants, quelling in fungi, and RNA interference in animals. Proc Natl Acad Sci U S A. 2000;97(21):11650–4. 10.1073/pnas.200217597 11016954 PMC17255

[19] Baumberger N, Baulcombe DC. Arabidopsis ARGONAUTE1 is an RNA Slicer that selectively recruits microRNAs and short interfering RNAs. Proc Natl Acad Sci U S A. 2005;102(33):11928–33. Epub 2005/08/04. 10.1073/pnas.0505461102 16081530 PMC1182554

[20] Iwakawa HO, Tomari Y. Molecular insights into microRNA-mediated translational repression in plants. Mol Cell. 2013;52(4):591–601. 10.1016/j.molcel.2013.10.033 . 24267452

[21] Carbonell A, Carrington JC. Antiviral roles of plant ARGONAUTES. Curr Opin Plant Biol. 2015;27:111–7. Epub 2015/07/17. 10.1016/j.pbi.2015.06.013 26190744 PMC4618181

[22] Ma Z, Zhang X. Actions of plant Argonautes: predictable or unpredictable? Curr Opin Plant Biol. 2018;45(Pt A):59–67. Epub 2018/05/29. 10.1016/j.pbi.2018.05.007 . 29857309

[23] Wylie SJ, Adams M, Chalam C, Kreuze J, López-Moya JJ, Ohshima K, et al ICTV Virus Taxonomy Profile: Potyviridae. J Gen Virol. 2017;98(3):352–4. 10.1099/jgv.0.000740 28366187 PMC5797945

[24] ICTV (2018). Virus Taxonomy: 2018 Release. The online (10th) Report of the International Committee on Taxonomy of Viruses EC 50. Washington, DC, July 2018.

[25] Rybicki EP. A Top Ten list for economically important plant viruses. Arch Virol. 2015;160(1):17–20. Epub 2014/11/29. 10.1007/s00705-014-2295-9 . 25430908

[26] Valli AA, Gallo A, Rodamilans B, López-Moya JJ, García JA. The HCPro from the Potyviridae family: an enviable multitasking Helper Component that every virus would like to have. Mol Plant Pathol. 2018;19(3):744–63. Epub 2017/05/26. 10.1111/mpp.12553 28371183 PMC6638112

[27] Pruss G, Ge X, Shi XM, Carrington JC, Bowman Vance V. Plant viral synergism: the potyviral genome encodes a broad-range pathogenicity enhancer that transactivates replication of heterologous viruses. Plant Cell. 1997;9(6):859–68. 10.1105/tpc.9.6.859 9212462 PMC156963

[28] Anandalakshmi R, Pruss GJ, Ge X, Marathe R, Mallory AC, Smith TH, et al A viral suppressor of gene silencing in plants. Proc Natl Acad Sci U S A. 1998;95(22):13079–84. 10.1073/pnas.95.22.13079 9789044 PMC23715

[29] Kasschau KD, Carrington JC. A counterdefensive strategy of plant viruses: suppression of posttranscriptional gene silencing. Cell. 1998;95(4):461–70. 10.1016/s0092-8674(00)81614-1 . 9827799

[30] Govier DA, Kassanis B, Pirone TP. Partial purification and characterization of the potato virus Y helper component. Virology. 1977;78(1):306–14. 10.1016/0042-6822(77)90101-5 . 860406

[31] Pirone TP, Blanc S. Helper-dependent vector transmission of plant viruses. Annu Rev Phytopathol. 1996;34:227–47. 10.1146/annurev.phyto.34.1.227 . 15012542

[32] Carrington JC, Herndon KL. Characterization of the potyviral HC-pro autoproteolytic cleavage site. Virology. 1992;187(1):308–15. 10.1016/0042-6822(92)90319-k . 1736533 PMC7173101

[33] Verchot J, Herndon KL, Carrington JC. Mutational analysis of the tobacco etch potyviral 35-kDa proteinase: identification of essential residues and requirements for autoproteolysis. Virology. 1992;190(1):298–306. 10.1016/0042-6822(92)91216-h . 1529535

[34] Valli A, Gallo A, Calvo M, de Jesús Pérez J, García JA. A novel role of the potyviral helper component proteinase contributes to enhance the yield of viral particles. J Virol. 2014;88(17):9808–18. Epub 2014/06/18. 10.1128/JVI.01010-14 24942578 PMC4136352

[35] Gallo A, Valli A, Calvo M, García JA. A Functional Link between RNA Replication and Virion Assembly in the Potyvirus. J Virol. 2018;92(9). Epub 2018/04/13. 10.1128/JVI.02179-17 29444942 PMC5899180

[36] Kasschau KD, Cronin S, Carrington JC. Genome amplification and long-distance movement functions associated with the central domain of tobacco etch potyvirus helper component-proteinase. Virology. 1997;228(2):251–62. 10.1006/viro.1996.8368 . 9123832

[37] Rojas MR, Zerbini FM, Allison RF, Gilbertson RL, Lucas WJ. Capsid protein and helper component-proteinase function as potyvirus cell-to-cell movement proteins. Virology. 1997;237(2):283–95. 10.1006/viro.1997.8777 . 9356340

[38] Sáenz P, Salvador B, Simón-Mateo C, Kasschau KD, Carrington JC, García JA. Host-specific involvement of the HC protein in the long-distance movement of potyviruses. J Virol. 2002;76(4):1922–31. 10.1128/jvi.76.4.1922-1931.2002 11799187 PMC135908

[39] Hafren A, Lohmus A, Makinen K. Formation of Potato Virus A-Induced RNA Granules and Viral Translation Are Interrelated Processes Required for Optimal Virus Accumulation. Plos Pathogens. 2015;11(12). 10.1371/journal.ppat.1005314 WOS:000368332800034. 26641460 PMC4671561

[40] Harvey JJ, Lewsey MG, Patel K, Westwood J, Heimstädt S, Carr JP, et al An antiviral defense role of AGO2 in plants. PLoS One. 2011;6(1):e14639 Epub 2011/01/31. 10.1371/journal.pone.0014639 21305057 PMC3031535

[41] Cao M, Du P, Wang X, Yu Y-Q, Qiu Y-H, Li W, et al Virus-activated plant siRNAs produced by RDR1. Proceedings of the National Academy of Sciences. 2014;111 (40):14613–8. 10.1073/pnas.1407131111 PMC4209997 25201959

[42] Garcia-Ruiz H, Carbonell A, Hoyer JS, Fahlgren N, Gilbert KB, Takeda A, et al Roles and programming of Arabidopsis ARGONAUTE proteins during Turnip mosaic virus infection. PLoS Pathog. 2015;11(3):e1004755 Epub 2015/03/25. 10.1371/journal.ppat.1004755 25806948 PMC4373807

[43] Fátyol K, Ludman M, Burgyán J. Functional dissection of a plant Argonaute. Nucleic Acids Res. 2016;44(3):1384–97. Epub 2015/12/15. 10.1093/nar/gkv1371 26673719 PMC4756824

[44] Zhang X, Yuan YR, Pei Y, Lin SS, Tuschl T, Patel DJ, et al Cucumber mosaic virus-encoded 2b suppressor inhibits Arabidopsis Argonaute1 cleavage activity to counter plant defense. Genes Dev. 2006;20(23):3255–68. 10.1101/gad.1495506 17158744 PMC1686603

[45] Baumberger N, Tsai CH, Lie M, Havecker E, Baulcombe DC. The Polerovirus silencing suppressor P0 targets ARGONAUTE proteins for degradation. Curr Biol. 2007;17(18):1609–14. 10.1016/j.cub.2007.08.039 . 17869110

[46] Bortolamiol D, Pazhouhandeh M, Marrocco K, Genschik P, Ziegler-Graff V. The Polerovirus F box protein P0 targets ARGONAUTE1 to suppress RNA silencing. Curr Biol. 2007;17(18):1615–21. 10.1016/j.cub.2007.07.061 . 17869109

[47] Azevedo J, Garcia D, Pontier D, Ohnesorge S, Yu A, Garcia S, et al Argonaute quenching and global changes in Dicer homeostasis caused by a pathogen-encoded GW repeat protein. Genes Dev. 2010;24(9):904–15. 10.1101/gad.1908710 20439431 PMC2861190

[48] Chiu MH, Chen IH, Baulcombe DC, Tsai CH. The silencing suppressor P25 of Potato virus X interacts with Argonaute1 and mediates its degradation through the proteasome pathway. Mol Plant Pathol. 2010;11(5):641–9. 10.1111/j.1364-3703.2010.00634.x 20696002 PMC6640501

[49] Giner A, Lakatos L, García-Chapa M, López-Moya JJ, Burgyán J. Viral protein inhibits RISC activity by argonaute binding through conserved WG/GW motifs. PLoS Pathog. 2010;6(7):e1000996 Epub 2010/07/15. 10.1371/journal.ppat.1000996 20657820 PMC2904775

[50] Csorba T, Kontra L, Burgyán J. viral silencing suppressors: Tools forged to fine-tune host-pathogen coexistence. Virology. 2015;479–480:85–103. Epub 2015/03/09. 10.1016/j.virol.2015.02.028 . 25766638

[51] Derrien B, Baumberger N, Schepetilnikov M, Viotti C, Cillia JD, Ziegler-Graff V, et al Degradation of AGO1 via autophagy. Proceedings of the National Academy of Sciences. 2012;109 (39):15942–6. 10.1073/pnas.1209487109 PMC3465452 23019378

[52] Fusaro AF, Correa RL, Nakasugi K, Jackson C, Kawchuk L, Vaslin MF, et al The Enamovirus P0 protein is a silencing suppressor which inhibits local and systemic RNA silencing through AGO1 degradation. Virology. 2012;426(2):178–87. Epub 2012/02/22. 10.1016/j.virol.2012.01.026 . 22361475

[53] Karran RA, Sanfaçon H. Tomato ringspot virus coat protein binds to ARGONAUTE 1 and suppresses the translation repression of a reporter gene. Mol Plant Microbe Interact. 2014;27(9):933–43. 10.1094/MPMI-04-14-0099-R . 24804809

[54] Ivanov KI, Eskelin K, Bašić M, De S, Lõhmus A, Varjosalo M, et al Molecular insights into the function of the viral RNA silencing suppressor HCPro. Plant J. 2016;85(1):30–45. 10.1111/tpj.13088 . 26611351

[55] Eskelin K, Suntio T, Hyvärinen S, Hafren A, Mäkinen K. Renilla luciferase-based quantitation of Potato virus A infection initiated with Agrobacterium infiltration of N. benthamiana leaves. J Virol Methods. 2010;164(1–2):101–10. Epub 2009/12/21. 10.1016/j.jviromet.2009.12.006 . 20026122

[56] Karlowski WM, Zielezinski A, Carrère J, Pontier D, Lagrange T, Cooke R. Genome-wide computational identification of WG/GW Argonaute-binding proteins in Arabidopsis. Nucleic Acids Res. 2010;38(13):4231–45. Epub 2010/03/24. 10.1093/nar/gkq162 20338883 PMC2910046

[57] Zielezinski A, Karlowski WM. Agos—a universal web tool for GW Argonaute-binding domain prediction. Bioinformatics. 2011;27(9):1318–9. Epub 2011/03/08. 10.1093/bioinformatics/btr128 . 21385787

[58] Hipper C, Brault V, Ziegler-Graff V, Revers F. Viral and cellular factors involved in Phloem transport of plant viruses. Front Plant Sci. 2013;4:154 Epub 2013/05/24. 10.3389/fpls.2013.00154 23745125 PMC3662875

[59] Pérez-Cañamás M, Hernández C. Key importance of small RNA binding for the activity of a glycine-tryptophan (GW) motif-containing viral suppressor of RNA silencing. J Biol Chem. 2015;290(5):3106–20. Epub 2014/12/10. 10.1074/jbc.M114.593707 25505185 PMC4317005

[60] Ludman M, Burgyán J, Fátyol K. Crispr/Cas9 Mediated Inactivation of Argonaute 2 Reveals its Differential Involvement in Antiviral Responses. Sci Rep. 2017;7(1):1010 Epub 2017/04/21. 10.1038/s41598-017-01050-6 28432338 PMC5430636

[61] Allen E, Xie Z, Gustafson AM, Carrington JC. microRNA-directed phasing during trans-acting siRNA biogenesis in plants. Cell. 2005;121(2):207–21. 10.1016/j.cell.2005.04.004 . 15851028

[62] Wang XB, Jovel J, Udomporn P, Wang Y, Wu Q, Li WX, et al The 21-nucleotide, but not 22-nucleotide, viral secondary small interfering RNAs direct potent antiviral defense by two cooperative argonautes in Arabidopsis thaliana. Plant Cell. 2011;23(4):1625–38. Epub 2011/04/05. 10.1105/tpc.110.082305 21467580 PMC3101545

[63] Eystathioy T, Chan EK, Tenenbaum SA, Keene JD, Griffith K, Fritzler MJ. A phosphorylated cytoplasmic autoantigen, GW182, associates with a unique population of human mRNAs within novel cytoplasmic speckles. Mol Biol Cell. 2002;13(4):1338–51. 10.1091/mbc.01-11-0544 11950943 PMC102273

[64] Till S, Lejeune E, Thermann R, Bortfeld M, Hothorn M, Enderle D, et al A conserved motif in Argonaute-interacting proteins mediates functional interactions through the Argonaute PIWI domain. Nat Struct Mol Biol. 2007;14(10):897–903. Epub 2007/09/23. 10.1038/nsmb1302 . 17891150

[65] El-Shami M, Pontier D, Lahmy S, Braun L, Picart C, Vega D, et al Reiterated WG/GW motifs form functionally and evolutionarily conserved ARGONAUTE-binding platforms in RNAi-related components. Genes Dev. 2007;21(20):2539–44. 10.1101/gad.451207 17938239 PMC2000319

[66] Zielezinski A, Karlowski WM. Early origin and adaptive evolution of the GW182 protein family, the key component of RNA silencing in animals. RNA Biol. 2015;12(7):761–70. 10.1080/15476286.2015.1051302 26106978 PMC4615383

[67] Del Toro FJ, Donaire L, Aguilar E, Chung BN, Tenllado F, Canto T. Potato Virus Y HCPro Suppression of Antiviral Silencing in Nicotiana benthamiana Plants Correlates with Its Ability To Bind. J Virol. 2017;91(12). Epub 2017/05/26. 10.1128/JVI.00367-17 28381573 PMC5446643

[68] Plisson C, Drucker M, Blanc S, German-Retana S, Le Gall O, Thomas D, et al Structural characterization of HC-Pro, a plant virus multifunctional protein. J Biol Chem. 2003;278(26):23753–61. Epub 2003/04/08. 10.1074/jbc.M302512200 . 12684502

[69] Urcuqui-Inchima S, Maia IG, Arruda P, Haenni AL, Bernardi F. Deletion mapping of the potyviral helper component-proteinase reveals two regions involved in RNA binding. Virology. 2000;268(1):104–11. 10.1006/viro.1999.0156 . 10683332

[70] Shiboleth YM, Haronsky E, Leibman D, Arazi T, Wassenegger M, Whitham SA, et al The conserved FRNK box in HC-Pro, a plant viral suppressor of gene silencing, is required for small RNA binding and mediates symptom development. J Virol. 2007;81(23):13135–48. Epub 2007/09/26. 10.1128/JVI.01031-07 17898058 PMC2169133

[71] Kasschau KD, Carrington JC. Long-distance movement and replication maintenance functions correlate with silencing suppression activity of potyviral HC-Pro. Virology. 2001;285(1):71–81. 10.1006/viro.2001.0901 . 11414807

[72] Cronin S, Verchot J, Haldeman-Cahill R, Schaad MC, Carrington JC. Long-distance movement factor: a transport function of the potyvirus helper component proteinase. Plant Cell. 1995;7(5):549–59. 10.1105/tpc.7.5.549 7780307 PMC160803

[73] Shi XM, Miller H, Verchot J, Carrington JC, Vance VB. Mutations in the region encoding the central domain of helper component-proteinase (HC-Pro) eliminate potato virus X/potyviral synergism. Virology. 1997;231(1):35–42. 10.1006/viro.1997.8488 . 9143300

[74] González-Jara P, Atencio FA, Martínez-García B, Barajas D, Tenllado F, Díaz-Ruíz JR. A Single Amino Acid Mutation in the Plum pox virus Helper Component-Proteinase Gene Abolishes Both Synergistic and RNA Silencing Suppression Activities. Phytopathology. 2005;95(8):894–901. 10.1094/PHYTO-95-0894 . 18944411

[75] Carrasco P, Daròs JA, Agudelo-Romero P, Elena SF. A real-time RT-PCR assay for quantifying the fitness of tobacco etch virus in competition experiments. J Virol Methods. 2007;139(2):181–8. Epub 2006/11/07. 10.1016/j.jviromet.2006.09.020 . 17092574

[76] Torres-Barceló C, Martín S, Daròs JA, Elena SF. From hypo- to hypersuppression: effect of amino acid substitutions on the RNA-silencing suppressor activity of the Tobacco etch potyvirus HC-Pro. Genetics. 2008;180(2):1039–49. Epub 2008/09/09. 10.1534/genetics.108.091363 18780745 PMC2567354

[77] Mingot A, Valli A, Rodamilans B, San León D, Baulcombe DC, García JA, et al The P1N-PISPO trans-Frame Gene of Sweet Potato Feathery Mottle Potyvirus Is Produced during Virus Infection and Functions as an RNA Silencing Suppressor. J Virol. 2016;90(7):3543–57. Epub 2016/01/20. 10.1128/JVI.02360-15 26792740 PMC4794657

[78] Feng L, Duan CG, Guo HS. Inhibition of in vivo Slicer activity of Argonaute protein 1 by the viral 2b protein independent of its dsRNA-binding function. Mol Plant Pathol. 2013;14(6):617–22. Epub 2013/04/28. 10.1111/mpp.12033 23621279 PMC6638910

[79] Cotton S, Grangeon R, Thivierge K, Mathieu I, Ide C, Wei T, et al Turnip mosaic virus RNA replication complex vesicles are mobile, align with microfilaments, and are each derived from a single viral genome. J Virol. 2009;83(20):10460–71. Epub 2009/08/05. 10.1128/JVI.00819-09 19656892 PMC2753101

[80] Grangeon R, Jiang J, Wan J, Agbeci M, Zheng H, Laliberté JF. 6K2-induced vesicles can move cell to cell during turnip mosaic virus infection. Front Microbiol. 2013;4:351 Epub 2013/12/04. 10.3389/fmicb.2013.00351 24409170 PMC3885808

[81] Wan J, Cabanillas DG, Zheng H, Laliberté JF. Turnip mosaic virus moves systemically through both phloem and xylem as membrane-associated complexes. Plant Physiol. 2015;167(4):1374–88. Epub 2015/02/25. 10.1104/pp.15.00097 25717035 PMC4378181

[82] Besong-Ndika J, Ivanov KI, Hafrèn A, Michon T, Mäkinen K. Cotranslational coat protein-mediated inhibition of potyviral RNA translation. J Virol. 2015;89(8):4237–48. Epub 2015/01/28. 10.1128/JVI.02915-14 25631087 PMC4442359

[83] Torrance L, Andreev IA, Gabrenaite-Verhovskaya R, Cowan G, Mäkinen K, Taliansky ME. An unusual structure at one end of potato potyvirus particles. J Mol Biol. 2006;357(1):1–8. Epub 2005/12/21. 10.1016/j.jmb.2005.12.021 . 16414068

[84] Cowan GH, Torrance L, Reavy B. Detection of potato mop-top virus capsid readthrough protein in virus particles. J Gen Virol. 1997;78 (Pt 7):1779–83. 10.1099/0022-1317-78-7-1779 . 9225055

[85] Peremyslov VV, Andreev IA, Prokhnevsky AI, Duncan GH, Taliansky ME, Dolja VV. Complex molecular architecture of beet yellows virus particles. Proc Natl Acad Sci U S A. 2004;101(14):5030–5. Epub 2004/03/25. 10.1073/pnas.0400303101 15044703 PMC387368

[86] Kiselyova OI, Yaminsky IV, Karpova OV, Rodionova NP, Kozlovsky SV, Arkhipenko MV, et al AFM study of potato virus X disassembly induced by movement protein. J Mol Biol. 2003;332(2):321–5. 10.1016/s0022-2836(03)00835-0 . 12948484

[87] Gabrenaite-Verkhovskaya R, Andreev IA, Kalinina NO, Torrance L, Taliansky ME, Mäkinen K. Cylindrical inclusion protein of potato virus A is associated with a subpopulation of particles isolated from infected plants. J Gen Virol. 2008;89(Pt 3):829–38. 10.1099/vir.0.83406-0 . 18272775

[88] Edelheit O, Hanukoglu A, Hanukoglu I. Simple and efficient site-directed mutagenesis using two single-primer reactions in parallel to generate mutants for protein structure-function studies. BMC Biotechnol. 2009;9:61 Epub 2009/06/30. 10.1186/1472-6750-9-61 19566935 PMC2711942

[89] Beauchemin C, Bougie V, Laliberté JF. Simultaneous production of two foreign proteins from a polyvirus-based vector. Virus Res. 2005;112(1–2):1–8. Epub 2005/04/13. 10.1016/j.virusres.2005.03.001 . 16022896

[90] Cui X, Yaghmaiean H, Wu G, Wu X, Chen X, Thorn G, et al The C-terminal region of the Turnip mosaic virus P3 protein is essential for viral infection via targeting P3 to the viral replication complex. Virology. 2017;510:147–55. Epub 2017/07/20. 10.1016/j.virol.2017.07.016 . 28735115

[91] Sánchez F, Martínez-Herrera D, Aguilar I, Ponz F. Infectivity of turnip mosaic potyvirus cDNA clones and transcripts on the systemic host Arabidopsis thaliana and local lesion hosts. Virus Res. 1998;55(2):207–19. 10.1016/s0168-1702(98)00049-5 . 9725673

[92] Nakagawa T, Kurose T, Hino T, Tanaka K, Kawamukai M, Niwa Y, et al Development of series of gateway binary vectors, pGWBs, for realizing efficient construction of fusion genes for plant transformation. J Biosci Bioeng. 2007;104(1):34–41. 10.1263/jbb.104.34 . 17697981

[93] Ivanov KI, Bašić M, Varjosalo M, Mäkinen K. One-step purification of twin-strep-tagged proteins and their complexes on strep-tactin resin cross-linked with bis(sulfosuccinimidyl) suberate (BS3). J Vis Exp. 2014;(86). Epub 2014/04/20. 10.3791/51536 24796313 PMC4174720

[94] Lõhmus A, Varjosalo M, Mäkinen K. Protein composition of 6K2-induced membrane structures formed during Potato virus A infection. Mol Plant Pathol. 2016;17(6):943–58. Epub 2016/02/17. 10.1111/mpp.12341 . 26574906 PMC6638329

