# Supplementary figures and images for: The potyviral silencing suppressor HCPro recruits and employs host ARGONAUTE1 in pro-viral functions
Source: PLoS Pathog. 2020 Oct 8;16(10):e1008965. doi: 10.1371/journal.ppat.1008965 (PMC7575100; doi:10.1371/journal.ppat.1008965)

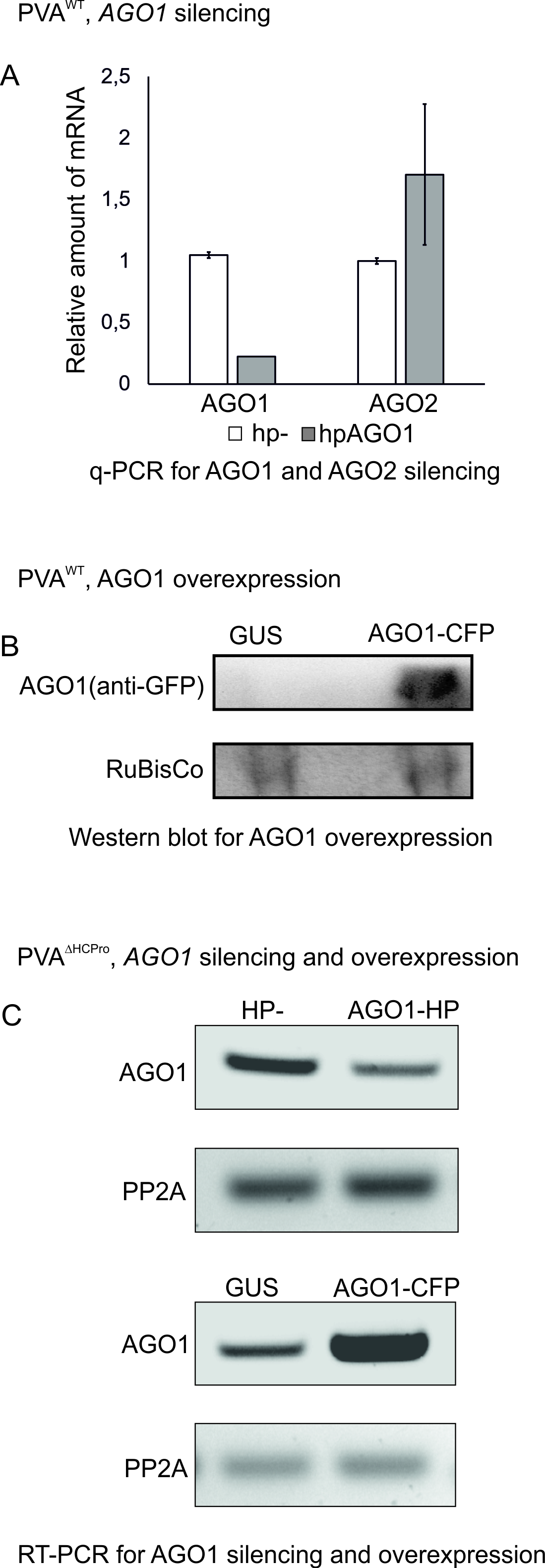

Supplement: S1 Fig — (A) Validation of the AGO1 silencing in mRNA level in Fig 1A–1C. Demonstrated is also upregulation in Ago2 mRNA due to AGO1 silencing. (B) AGO1-CFP over-expression corresponding to Fig 1D–1F verified by α-CFP western blot. Ponceau-S staining of RuBisCo shows equivalent loading (C) Validation of AGO1 silencing (upper panel) and over-expression (lower panel), related to Fig 1G and 1H, by RT-PCR at mRNA level. The mRNA levels of the housekeeping gene PP2A served as a loading control. (TIF) [file ppat.1008965.s001.tif]

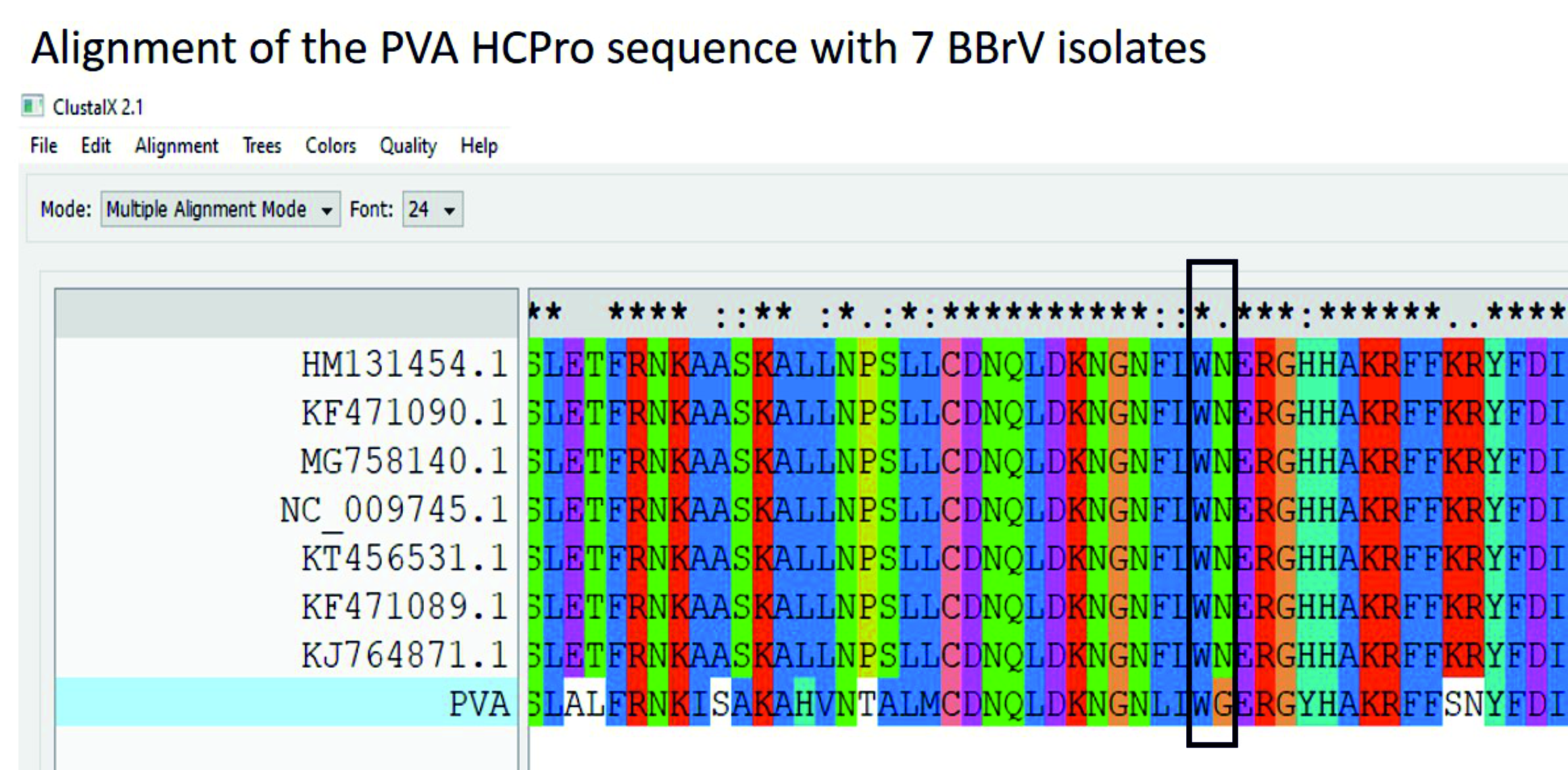

Supplement: S2 Fig — A comparison of HCPro sequences of seven BBrMV isolates with PVA HCPro revealed the absence of WG motif in BBrMV. ‘W’ is in the same position as in other potyviruses but ‘G’ is substituted by ‘N’ in this virus. (TIF) [file ppat.1008965.s002.tif]

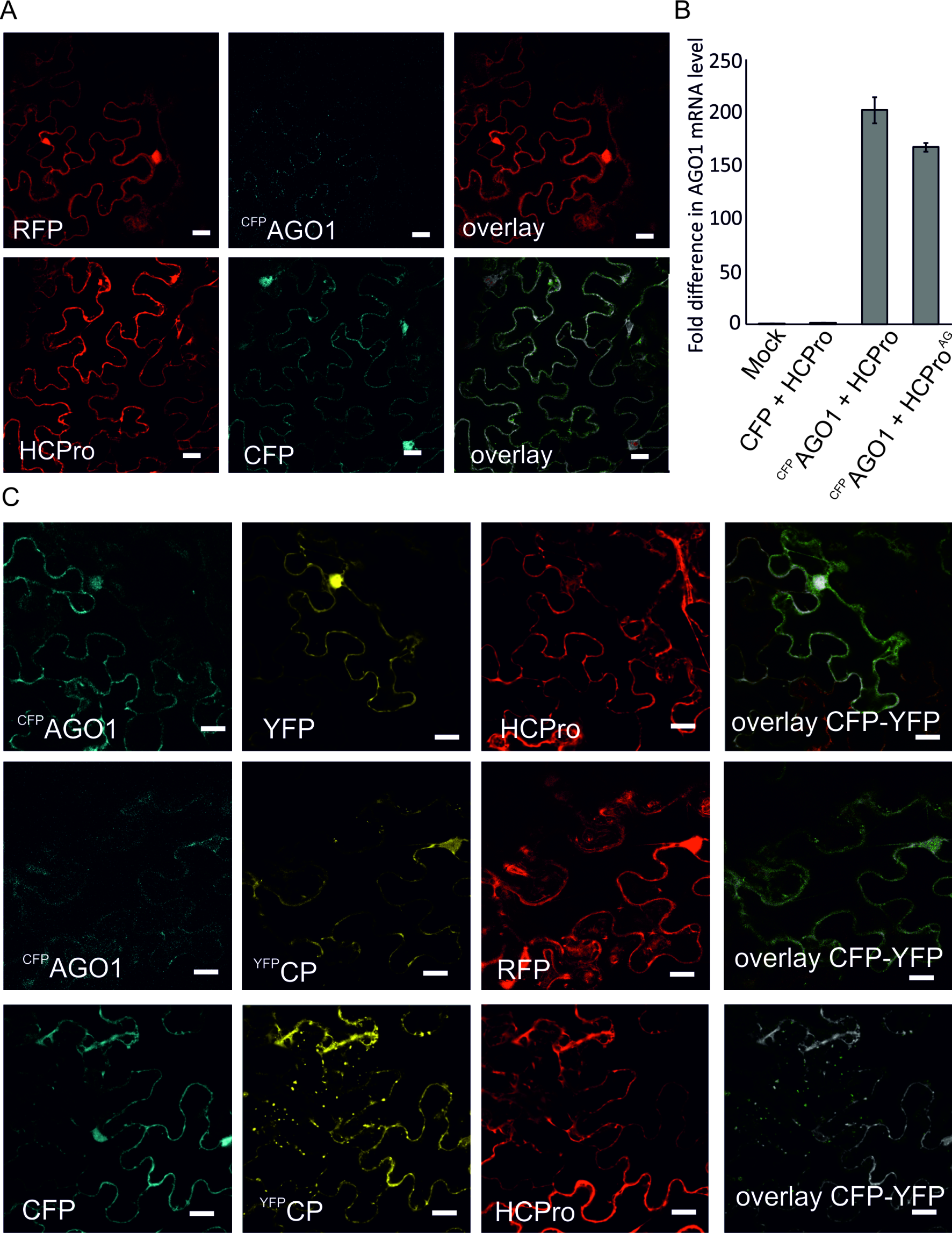

Supplement: S3 Fig — A) Control studies for co-localization of HCPro and HCProAG together with CFPAGO1 in N. benthamiana leaves. Unfused RFP was used as a control for HCPro/HCProAG and CFP for CFPAGO1. All constructs were agroinfiltrated at OD600 0.1 and leaves were imaged by confocal microscopy at 3 dpi. B) Validation of the AGO1CFP expression in mRNA level in Fig 4B overexpression C) Control studies for co-localization of YFPCP together with CFPAGO1 and HCPro/HCProAG. Unfused RFP was used as a control for HCPro/HCProAG, YFP for YFPCP and CFP for CFPAGO1. Representative images from three independent experiments are shown, scale bar is 20 μm. (TIF) [file ppat.1008965.s003.tif]

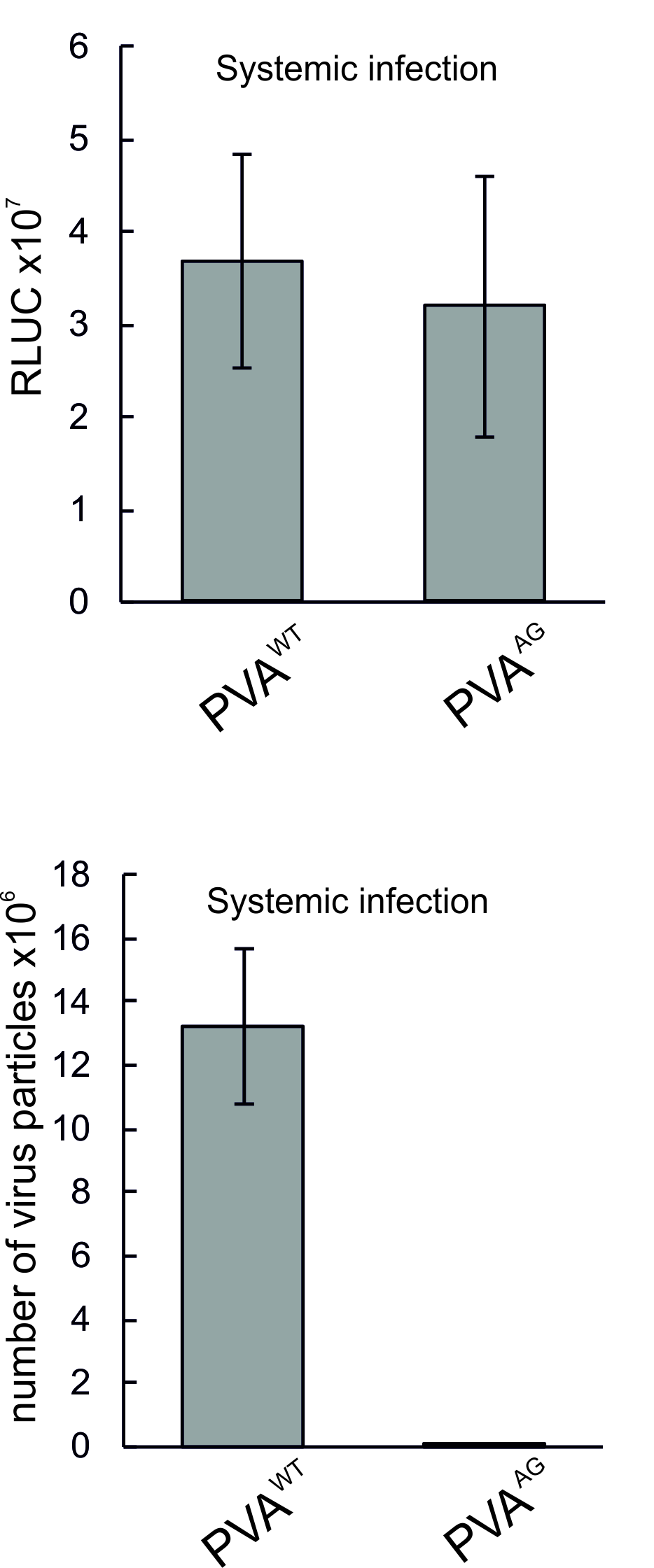

Supplement: S4 Fig — PVAWT and PVAAG expression level determined by the RLUC assay (upper panel) and particle amounts determined by IC-RT-PCR (lower panel) corresponding to the EM images shown in Fig 7. (TIF) [file ppat.1008965.s004.tif]

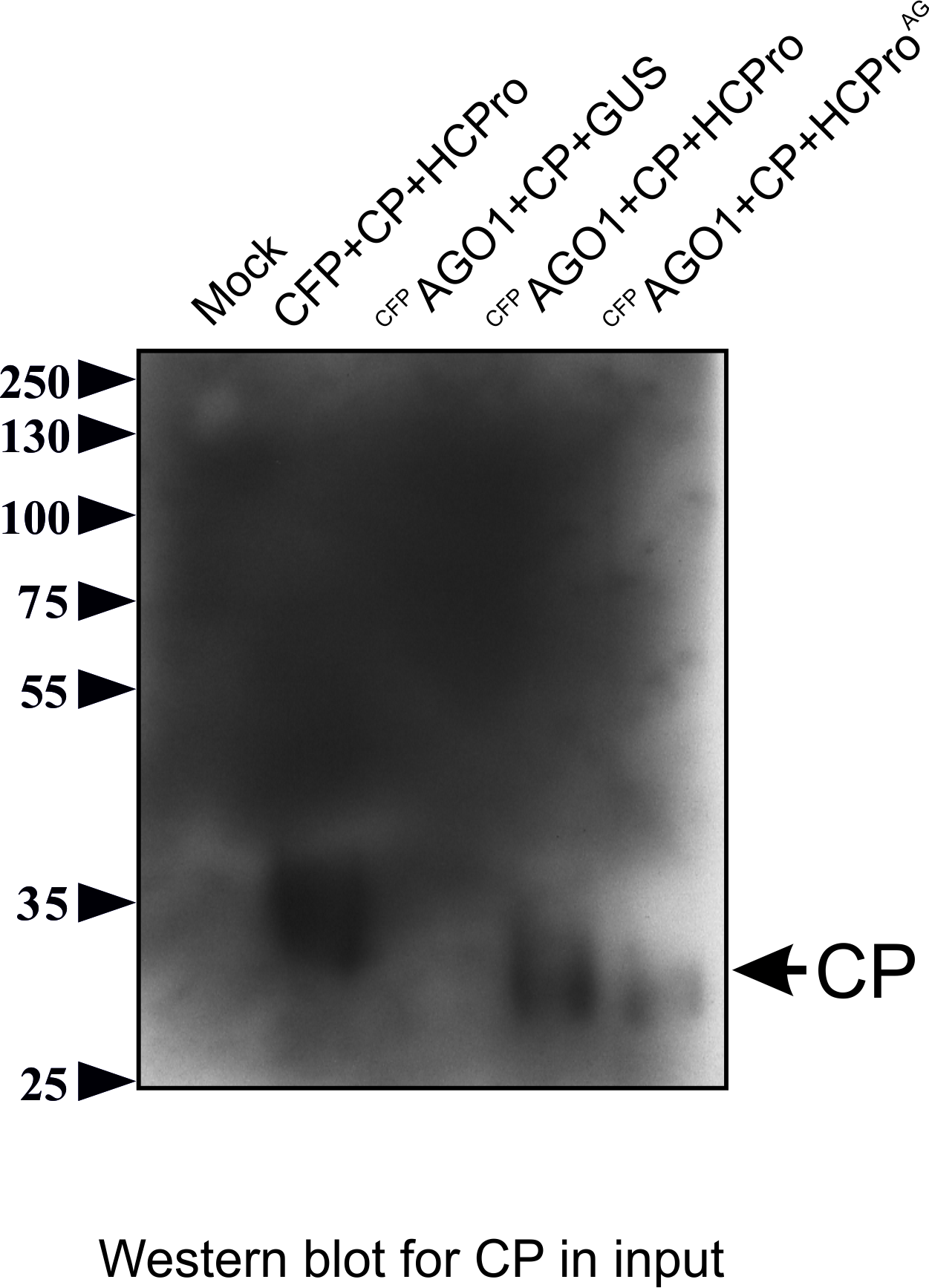

Supplement: S5 Fig — (TIF) [file ppat.1008965.s005.tif]

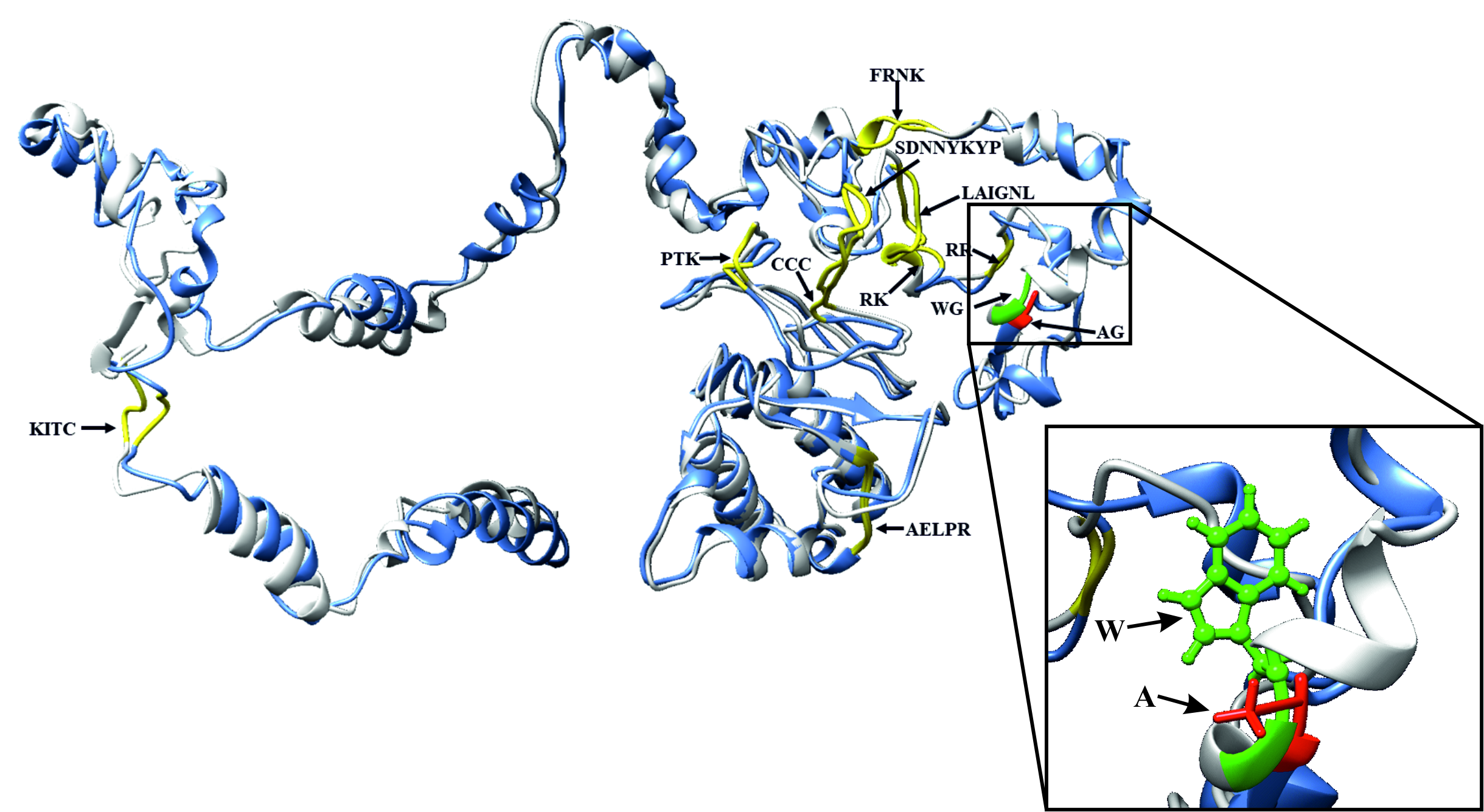

Supplement: S6 Fig — Structural predictions of HCProWT (in white) and HCProAG (in blue) were independently made using I-TASSER protein prediction tool and server. Models consistent to TuMV HCPro crystal structure (PDB id-3RNV) were overlaid using Chimera protein structure visualization software. Both the structures matched well and positions of several important functional motifs of HCPro (in yellow) were intact. Finally, WG (in green) and its mutated counterpart AG (in red) were also found to be in close vicinity, however, their molecular structure varied as amino acid ‘W’ was replaced with ‘A’ (in the box). (TIF) [file ppat.1008965.s006.tif]
