# Supplementary material for: The potyviral silencing suppressor HCPro recruits and employs host ARGONAUTE1 in pro-viral functions
Source: PLoS Pathog. 2020 Oct 8;16(10):e1008965. doi: 10.1371/journal.ppat.1008965 (PMC7575100; doi:10.1371/journal.ppat.1008965)
Supplement: S1 Table — Related to Fig 2. (DOCX) [file ppat.1008965.s007.docx]

Table S1. Conservation of the WG motif in 113 potyviral HCPros. Related to Figure 2.

| Potyvirus species, accession no./amino acid range | Amino acid sequence |
| --- | --- |
| *Algerian watermelon mosaic virus, YP_001931956.1/610-623* | NFL**WG**DRQYHAKRF |
| *Apium virus Y, YP_004123951.1/614-627* | NFL**WG**QRGYHAKRF |
| *Arracacha mottle virus, YP_006522440.1/551-564* | GFI**WG**QREPHAKRF |
| *Asparagus virus 1, YP_009110712.1/539-552* | NFV**WG**KRGYHAKRF |
| *Banana bract mosaic virus, YP_001427389.1/534-547* | NFLWNERGHHAKRF |
| *Barbacena virus Y, YP_009272820.1/491-504* | NFL**WG**EREYHARRF |
| *Basella rugose mosaic virus, YP_001427385.1/513-526* | NFI**WG**QRSYHARRF |
| *Bean common mosaic necrosis virus, NP_660175.1/522-535* | NFV**WG**ERGRHSKRF |
| *Bean common mosaic virus, NP_570723.1/628-641* | NFI**WG**ERGYHSKRF |
| *Bean yellow mosaic virus, NP_612218.1/489-502* | NFV**WG**QRAYHAKRF |
| *Beet mosaic virus, NP_954611.1/518-531* | NFI**WG**ERGYHSKRF |
| *Bidens mosaic virus, YP_008877631.1/488-501* | NFL**WG**QREYHAKRF |
| *Bidens mottle virus, YP_003778191.1/492-505* | SFI**WG**QREYHAKRI |
| *Blue squill virus A, YP_006990202.1/522-535* | NFV**WG**ERGRHSKRF |
| *Brugmansia mosaic virus, YP_007354880.1/495-508* | NFL**WG**QREYHAKRF |
| *Brugmansia suaveolens mottle virus, YP_003900466.1/503-516* | NFL**WG**QREYHAKRF |
| *Calla lily latent virus, YP_007969411.1/534-547* | NFV**WG**ERAKHSKRF |
| *Callistephus mottle virus, YP_009272570.1/582-595* | NFK**WG**KRGYHAKRF |
| *Canna yellow streak virus, YP_003208047.1/447-460* | NFL**WG**QRAYHAKRF |
| *Carrot thin leaf virus, YP_009091824.1/504-517* | NFK**WG**ERAYHAKRF |
| *Catharanthus mosaic virus, YP_009143308.1/501-514* | NFL**WG**KRGYHAKRL |
| *Celery mosaic virus, YP_004376199.1/606-619* | NFL**WG**QRGYHAKRF |
| *Chilli ringspot virus, YP_004875339.1/514-527* | NFV**WG**KRGYHAKRF |
| *Chilli veinal mottle virus, NP_982308.1/505-518* | NFI**WG**KRGYHAKRF |
| *Clover yellow vein virus, NP_613273.1/507-520* | NFM**WG**QRAYHAKRF |
| *Cocksfoot streak virus, NP_620483.1/514-527* | NFV**WG**ERSHHAKRF |
| *Colombian datura virus, YP_007346986.1/517-530* | NFV**WG**ARGYHAKRF |
| *Cowpea aphid-borne mosaic virus, NP_659018.1/490-503* | NFI**WG**ERGRHSKRF |
| *Cyrtanthus elatus virus A, YP_006383504.1/522-535* | VFQ**WG**ERGYHAKRL |
| *Daphne virus Y, AMR93994.1/503-516* | NFV**WG**TRGYHARRF |
| *Dasheen mosaic virus, NP_613274.1/591-604* | NFV**WG**ERGYHSKRF |
| *Donkey orchid virus A, YP_007969412.1/621-634* | NFV**WG**QRGYQSLQI |
| *East Asian Passiflora virus, YP_459919.1/640-653* | NFV**WG**ERGKHSKRF |
| *Freesia mosaic virus, YP_003587807.1/502-515* | NFV**WG**ERGRHSKRF |
| *Fritillary virus Y, YP_001974419.1/515-528* | NFI**WG**ERGKHSKRF |
| *Gloriosa stripe mosaic virus, ABR88099.1/486-499* | NFI**WG**KRGYHAKRF |
| *Habenaria mosaic virus, YP_008240477.1/467-480* | NFI**WG**DRAYHAKRF |
| *Hardenbergia mosaic virus, YP_004564588.1/517-530* | NFI**WG**ERGRHSKRF |
| *Hippeastrum mosaic virus, YP_006382256.1/535-548* | NFV**WG**LRGAAVNRF |
| *Hyacinth mosaic virus, YP_009465708.1/533-546* | NFL**WG**ERGYHAKRF |
| *Impatiens flower break virus, YP_009255234.1/523-536* | NFI**WG**ERGYHSKRV |
| *Iranian johnsongrass mosaic virus, YP_006906026.1/441-454* | NFL**WG**RREYHAKRF |
| *Iris severe mosaic virus, YP_009224125.1/647-660* | VFQ**WG**ERGVHAKRF |
| *Japanese yam mosaic virus, NP_051161.1/533-546* | NFV**WG**ERGYHAKRF |
| *Jasmine virus T, YP_009221980.1/501-514* | NFV**WG**LRDTHAKRF |
| *Johnsongrass mosaic virus, NP_619668.1/446-459* | DFL**WG**QRAYHAKRF |
| *Keunjorong mosaic virus, YP_004934107.1/514-527* | NFI**WG**ERGYHSKRL |
| *Konjac mosaic virus, YP_529485.1/532-545* | NFV**WG**ERSYHAKRF |
| *Leek yellow stripe virus, NP_659009.1/566-579* | NFI**WG**ERGYHAKRF |
| *Lettuce Italian necrotic virus, YP_009162372.1/604-617* | SFK**WG**KRQYHAKRF |
| *Lettuce mosaic virus, NP_619667.1/643-656* | GFE**WG**QRSYHAKRF |
| *Lily mottle virus, NP_945133.1/516-529* | NFE**WG**QRGYHAKRF |
| *Lupinus mosaic virus, YP_004123732.1/668-681* | NFV**WG**QRGYHAKRF |
| *Maize dwarf mosaic virus, NP_569138.1/441-454* | NFV**WG**KREYHAKRF |
| *Moroccan watermelon mosaic virus, YP_001552410.1/550-563* | NFL**WG**ERQYHAKRY |
| *Narcissus degeneration virus, YP_001019187.1/600-613* | VFQ**WG**DRGYHAKRI |
| *Narcissus late season yellows virus, YP_009010942.1/518-531* | NFI**WG**ERGYHAKRF |
| *Narcissus yellow stripe virus, YP_002308453.1/523-536* | NFV**WG**ERGYHAKRF |
| *Onion yellow dwarf virus, NP_871002.1/666-679* | IFQ**WG**ERGYHAKRF |
| *Ornithogalum mosaic virus, YP_006989380.1/469-482* | NFL**WG**KRGYHAKRF |
| *Panax virus Y, YP_003725718.1/510-523* | NFI**WG**QRGYHAKRF |
| *Papaya leaf distortion mosaic virus, NP_870995.1/687-700* | NFV**WG**ERGYHAKRF |
| *Papaya ringspot virus, NP_056758.1/752-765* | NFL**WG**ERQYHAKRF |
| *Passion fruit woodiness virus, YP_004063671.1/522-535* | NFI**WG**ERGYHSKRF |
| *Pea seed-borne mosaic virus, NP_056765.1/604-617* | NFV**WG**ERGYHAKRF |
| *Peanut mottle virus, NP_068348.2/527-540* | NFV**WG**ERGRHSKRF |
| *Pecan mosaic-associated virus, YP_009256204.1/400-413* | NFL**WG**QRGLHAKRF |
| *Pennisetum mosaic virus, YP_249455.1/454-467* | NFL**WG**KREYHAKRF |
| *Pepper mottle virus, NP_041276.1/491-504* | SFL**WG**QREYHARRF |
| *Pepper severe mosaic virus, YP_778468.1/495-508* | NFL**WG**QREYHARRF |
| *Pepper veinal mottle virus, YP_002519375.1/505-518* | NFV**WG**KRGYHAKRF |
| *Pepper yellow mosaic virus, YP_003778216.1/494-507* | NFL**WG**QREYHAKRF |
| *Peru tomato mosaic virus, NP_787937.1/493-506* | NFL**W**GQREYHAKRF |
| *Plum pox virus, CAB51641.1/514-527* | NFV**WG**QREYHAKRF |
| *Pokeweed mosaic virus, AFS28881.1/494-507* | NFL**WG**KREYHAKRF |
| *Potato virus A, NP_659729.1/503-516* | NLI**WG**ERGYHAKRF |
| *Potato virus V, MK756120.1* | NFL**WG**QREYHAKRF |
| *Potato virus Y, AAB50573.1/508-521* | NFL**WG**QREYHAKRF |
| *Scallion mosaic virus, NP_570725.1/416-429* | NFV**WG**ERGYHAKRF |
| *Shallot yellow stripe virus, YP_331412.1/676-689* | MFQ**WG**ERGYHAKGL |
| *Sorghum mosaic virus, NP_659391.1/441-454* | DFL**WG**KREYHAKRF |
| *Soybean mosaic virus, NP_072165.1/513-526* | NFV**WG**ERGRHSKRF |
| *Sugarcane mosaic virus, NP_570724.1/441-454* | NFV**WG**NREYHAKRF |
| *Sunflower chlorotic mottle virus, YP_003580192.1/616-629* | NFV**WG**QREYHAKRF |
| *Sunflower mild mosaic virus, YP_007872240.1/568-581* | NFM**WG**ERGYHAKRF |
| *Sunflower ring blotch virus, YP_009351870.1/488-501* | NFV**WG**QREYHAKRF |
| *Sweet potato feathery mottle virus, NP_045216.1/870-883* | NLI**WG**ERGYHSKRF |
| *Sweet potato latent virus, YP_007697620.1/648-661* | NFI**WG**ERGYHAKRF |
| *Sweet potato virus 2, YP_006382460.1/824-837* | NLI**WG**DRGYHSKRF |
| *Sweet potato virus C, YP_004046670.1/860-873* | NLI**WG**ERGYHSKRF |
| *Sweet potato virus G, AFM30943.1/824-837* | NLI**WG**ERGYHSKRF |
| *Tamarillo leaf malformation virus, YP_009126730.1/515-528* | NFV**WG**ERGYHAKRF |
| *Telosma mosaic virus, YP_001427386.1/522-535* | NFI**WG**ERSRHSKRF |
| *Thunberg fritillary mosaic virus, YP_254713.1/566-579* | NFL**WG**QRGYHAKRF |
| *Tobacco etch virus, NP_062908.1/511-524* | NFI**WG**LRGAHAKRF |
| *Tobacco mosqueado virus, YP_009252304.1/539-552* | NFI**WG**TREYHAKRF |
| *Tobacco vein banding mosaic virus, YP_001552409.1/514-527* | NFV**WG**KRGYHAKRF |
| *Tobacco vein mottling virus, NP_056867.1/479-492* | NFL**WG**LAGIAAKRF |
| *Tomato necrotic stunt virus, YP_006272948.1/526-539* | NFR**WG**QREYHAKRF |
| *Turnip mosaic virus, NP_062866.2/568-581* | NFI**WG**ERGYHAKRF |
| *Vanilla distortion mosaic virus, YP_009091808.1/508-521* | NFV**WG**ERGRHAKRF |
| *Watermelon mosaic virus, YP_077181.1/648-661* | NFV**WG**ERGKHSKRL |
| *Verbena virus Y, YP_001931955.1/489-502* | SFL**WG**QREYHAKRF |
| *Wild onion symptomless virus, YP_009259366.1/442-455* | NFI**WG**ERGYHAKRF |
| *Wild potato mosaic virus, NP_741959.1/493-506* | NFL**WG**QREYHAKRF |
| *Wild tomato mosaic virus, YP_001427388.1/503-516* | NFV**WG**KRGYHAKRF |
| *Wisteria vein mosaic virus, YP_271857.1/522-535* | NFV**WG**ERGRHSKRF |
| *Yam mild mosaic virus, YP_006990077.1/525-538* | NFK**WG**RRGYHAKRF |
| *Yam mosaic virus, YP_022751.1/503-516* | NFM**WG**QRGYHAKRF |
| *Zantedeschia mild mosaic virus, AAV54595.4/568-581* | NFV**WG**ARGYHSKRF |
| *Zucchini shoestring virus, ANH22633.1/778-791* | NFL**WG**DRQYHAKRF |
| *Zucchini tigre mosaic virus, AGY36217.1/748-761* | NFL**WG**ERQYHAKRF |
| *Zucchini yellow mosaic virus, AAD44684.2/514-527* | NFV**WG**ERGYHSKRL |
